# A manganese-sparing response balances competing cellular demands to enable *Staphylococcus aureus* infection

**DOI:** 10.1128/mbio.01439-25

**Published:** 2025-08-18

**Authors:** Riley A. McFarlane, Jana N. Radin, Rafał Mazgaj, Kevin J. Waldron, David Lalaouna, Thomas E. Kehl-Fie

**Affiliations:** 1 Department of Microbiology and Immunology, University of Iowa 311821 , Iowa City, Iowa, USA; 2 Institute of Biochemistry and Biophysics Polish Academy of Sciences, Warsaw, Poland; 3 Université de Strasbourg, CNRS, Architecture et Réactivité de l’ARN, UPR9002, Strasbourg, France; University of Pretoria, Pretoria, Gauteng, South Africa

**Keywords:** *Staphylococcus aureus*, RsaC, small RNA, metal limitation, manganese, oxidative stress, superoxide dismutase

## Abstract

Responding to stress is critical to the survival of life, especially for microbes that have a limited ability to manipulate their environment. During infection, *Staphylococcus aureus* and other invaders must overcome both the host-imposed absence of manganese and the oxidative burst of immune cells, which increases the need for this essential metal. The current investigations revealed that a small RNA, RsaC, integrates the staphylococcal responses to manganese starvation and oxidative stress. Upon manganese limitation, RsaC activates a manganese-sparing response, which decreases the cellular demand for manganese, enabling growth in manganese-restricted environments. However, the benefit of this response is environment-dependent as RsaC suppresses the expression of the manganese-dependent superoxide dismutase SodA, sensitizing *S. aureus* to oxidative stress. Despite this suppression, RsaC is necessary for *S. aureus* to cause infection, with its importance dependent on the efficacy of the host’s manganese withholding response. These results reveal a previously unappreciated manganese-sparing response that is important for bacterial virulence, and the imperative role of sRNAs in balancing bacterial adaptation to stressors that place conflicting demands on cellular physiology.

**IMPORTANCE:**

During infection, pathogens must utilize processes that impose conflicting cellular demands. This conflict is exemplified by the need of *Staphylococcus aureus* to preserve essential processes and survive the oxidative burst of immune cells, both of which require manganese despite experiencing host-imposed manganese starvation. The current investigations revealed that *S. aureus* activates a manganese-sparing response controlled by the regulatory RNA, RsaC, in response to host-imposed manganese starvation. This small RNA sacrifices the expression of a manganese-dependent superoxide dismutase to preserve the activity of essential manganese-dependent processes. Despite this, RsaC is necessary for infection, revealing the important role of this manganese-sparing response to pathogenesis and that invaders must actively compromise ideal stress responses to cause disease.

## INTRODUCTION

Responding to change is critical for the survival of life. This is particularly true for microbes, which have little control over their environment. Complicating this task, the stresses they encounter frequently impose conflicting demands on the cell (1). Exemplifying this challenge is metal limitation and oxidative stress (2). Metals are required for life, due to their involvement in essential cellular processes, including metabolism, signal transduction, and DNA replication (3). To defend against oxidative stress, organisms across the tree of life utilize superoxide dismutases (SOD). These enzymes also require a metal cofactor for function, and SOD activity is important for viability in the presence of superoxide (4). However, the use of these conditionally essential enzymes increases the cellular demand for metal (2, 5, 6). Thus, in metal-limited environments, microbes must balance maintaining the activity of essential metal-dependent processes with those that are conditionally essential. How microbes manage these conflicts and how these conflicts restrict the paths to survival available to an organism remain poorly understood. Within the human body, in association with plants and in the environment, microbes must confront multiple stressors simultaneously; therefore, how they compromise their ideal response will inform not only our ability to treat human disease but also inform agricultural and environmental endeavors that benefit humans.

Half of all enzymes require a metal cofactor, and the host leverages this dependency to combat pathogens by actively removing metals from sites of infection (3, 7, 8). This critical host defense, known as nutritional immunity, imposes metal starvation on invaders. Defects in nutritional immunity increase susceptibility to infection by fungal and bacterial pathogens in both experimental systems and humans (9–12). Metal starvation imposed by this defense inactivates microbial processes, thereby reducing growth and sensitizing invaders to other antimicrobial weapons wielded by the immune response (2). A prime example of nutritional immunity is the abscess formed by *S. aureus*, which is rendered virtually devoid of manganese (Mn) by the immune effector calprotectin (CP) (10). This metal binding protein can be found at sites of infection in excess of 1 mg/mL (13). Loss of CP increases metal availability during infection and sensitizes the host to infection by *S. aureus* and other pathogens (9, 10, 14). The susceptibility to infection in the absence of CP results from increased metal availability, which enables *S. aureu*s and other microbes to maintain the activity of metal-dependent enzymes (2).

The respiratory burst of phagocytes, which produces reactive oxygen species, is also a critical host defense (15, 16). SODs enable pathogens to survive the oxidative burst, but their reliance on a metal cofactor renders them susceptible to nutritional immunity (2). This susceptibility is exemplified by SodA from *S. aureus*, which is dependent on Mn for function and whose activity is reduced in Mn-limited environments, including in the presence of CP. Unlike most metalloenzymes, SODs bind their cofactor irreversibly (17), preventing redistribution to other enzymes as the needs of the cell change. As such, expression of these conditionally essential enzymes poses an increased challenge to cells experiencing metal limitation by further reducing the availability of metal cofactors for essential processes.

Efforts to understand bacterial adaptation to metal limitation have classically focused on proteinaceous regulators that directly sense cytosolic metal availability and alter transcription. However, it is now apparent that post-transcriptional regulation contributes to controlling the bacterial response to iron (Fe) availability, through the small RNA (sRNA) RyhB and analogs, enabling adaptation to Fe-limited environments (18–21). However, it is not known if sRNAs contribute to the ability of bacteria to survive in the absence of non-Fe metals, such as Mn, and to cause infection. An sRNA was recently identified, RsaC, that is encoded in the 3′ UTR of the *S. aureus* Mn transporter *mntABC*. RsaC is induced in response to Mn limitation and interacts with multiple transcripts (22, 23). The current work reveals that RsaC plays a critical role in enabling *S. aureus* to respond to nutritional immunity and cause infection, through its activation of a Mn-sparing response and its key role in balancing the activity of essential and conditionally essential processes during infection.

## RESULTS

### RsaC contributes to resisting calprotectin-imposed manganese starvation

**Fig 1:**
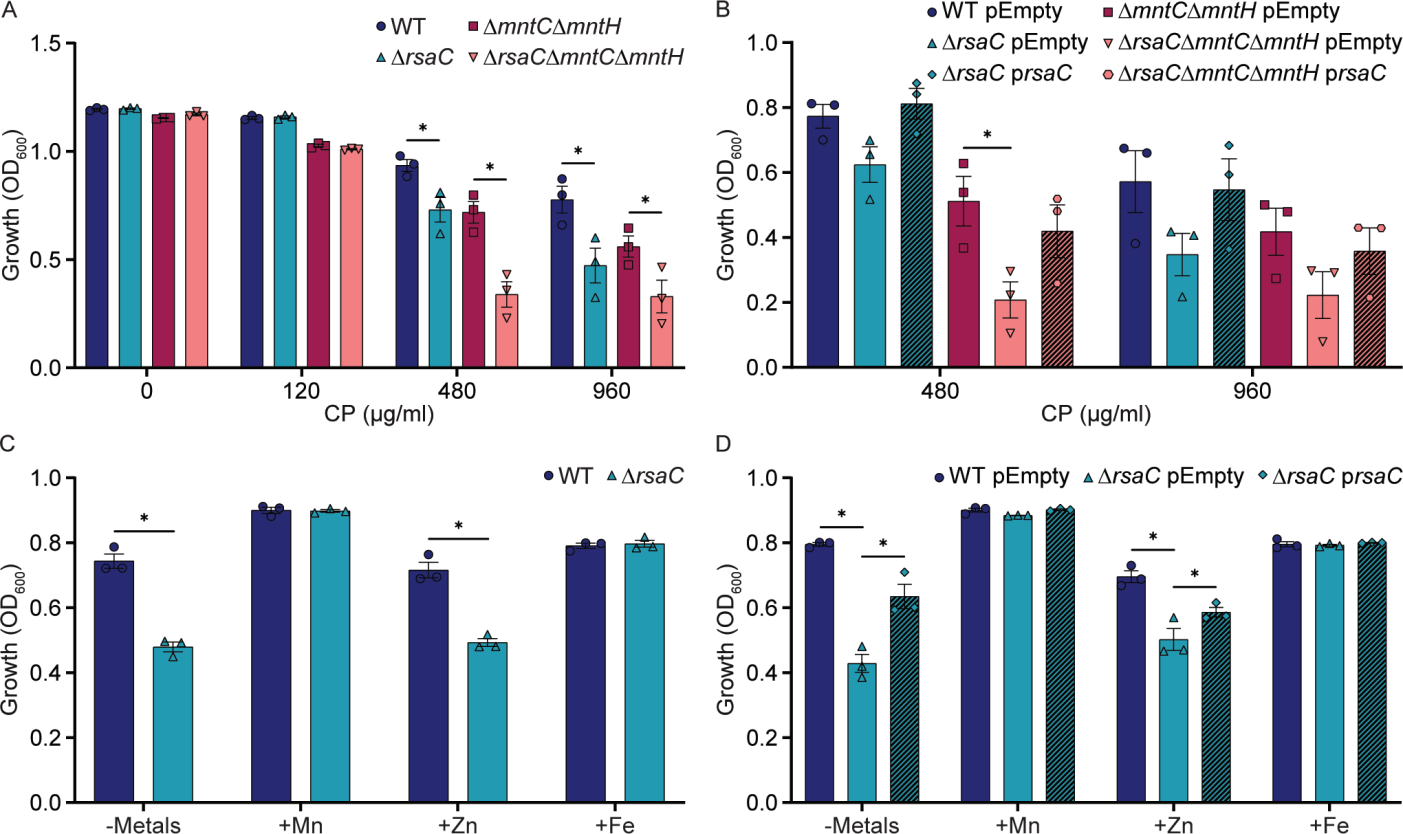
Loss of RsaC reduces the ability of *S. aureus* to grow in manganese-limited environments. (**A, B**) The indicated strains of *S. aureus* were incubated in defined medium containing glucose and amino acids as carbon sources in the presence of CP, and growth was determined by assessing optical density after 10 hours. (**C, D**) The indicated strains of *S. aureus* were incubated in metal-limited NRPMI supplemented with 1 µM MnCl_2_, ZnSO_4_, or FeSO_4_ as indicated, and growth was assessed after 8 hours by measuring optical density. (**B, D**) As indicated, the strains contain either an empty vector (pEmpty) or an RsaC-expressing plasmid (p*rsaC*). *P ≤ 0.05 for the indicated comparison was determined by two-way ANOVA with Šidák’s multiple comparisons test. *n* = 3. Error bars = SEM.

To test the hypothesis that RsaC enables *S. aureus* to overcome metal starvation, wild-type *S. aureus* and Δ*rsaC* were incubated in a defined medium containing glucose, amino acids, and CP, and growth was assessed. Loss of RsaC sensitized *S. aureus* to CP (Fig. 1A; Fig. S1A), while expression of *rsaC* from a plasmid reversed the growth defect of Δ*rsaC* (Fig. 1B; Fig. S1B). These results indicate that RsaC enables growth under host-imposed metal starvation. Next, the hypothesis that RsaC promotes resistance to host-imposed Mn starvation was tested. First, the ability of wild-type *S. aureus* and Δ*rsaC* to grow in a metal-defined medium, NRPMI (24), was evaluated. In a medium lacking Mn, Zn, and Fe, Δ*rsaC* grows less robustly than the wild type, with the defect reversed by the addition of Mn or Fe or by ectopic expression of *rsaC* from a plasmid (Fig. 1C and D; Fig. S1C and D). The rescue by Fe was surprising, as RsaC expression is controlled by Mn availability (Fig. S2) (22, 23). However, many enzymes are capable of using Fe or Mn for function (25), suggesting that Fe supplementation may mask the impact of Mn limitation. To further test the hypothesis that RsaC promotes resistance to Mn starvation, the impact of losing RsaC in a strain lacking both staphylococcal Mn transporters, Δ*mntC*Δ*mntH,* was evaluated. Loss of the Mn transporters exacerbated the growth defect observed with Δ*rsaC* in the presence of CP but was reversed when *rsaC* was expressed from a plasmid (Fig. 1A and B; Fig. S1A and B). Furthermore, the RsaC mutants were not sensitive to a CP variant unable to bind Mn (Δ6His) (Fig. S3A) (26). Together, these observations indicate that RsaC protects against Mn starvation imposed by a critical immune effector.

### RsaC enables metabolic flexibility when manganese starved

**Fig 2:**
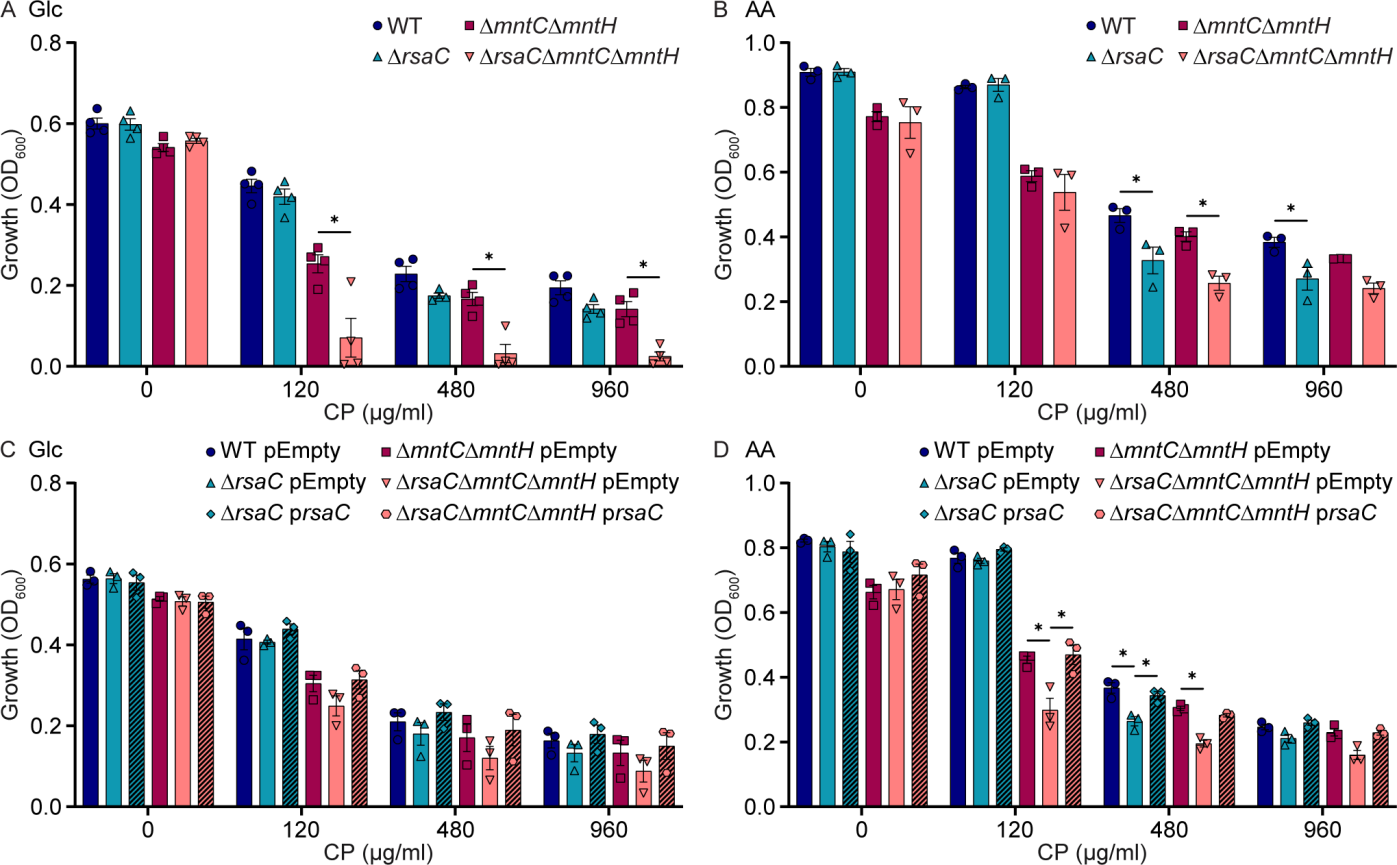
RsaC enables the use of both glucose and amino acids when manganese-starved. The indicated strains of *S. aureus* were incubated in defined medium containing either (**A, C**) glucose (Glc) or (**B, D**) amino acids (AA) as the sole carbon source in the presence of CP, and growth was assessed by evaluating optical density after 10 hours. (**C, D**) As indicated, the strains contain either an empty vector (pEmpty) or an RsaC-expressing plasmid (p*rsaC*). *P ≤ 0.05 for the indicated comparison was determined by two-way ANOVA with Šidák’s multiple comparisons test. *n* ≥ 3. Error bars = SEM.

Metabolic flexibility is critical to the success of *S. aureus* as a pathogen, with the ability to consume both glucose and amino acids contributing to infection (1, 27, 28). To test the hypothesis that RsaC contributes to utilization of both energy sources, the growth of wild-type *S. aureus*, Δ*rsaC,* Δ*mntC*Δ*mntH*, and Δ*rsaC*Δ*mntC*Δ*mntH* was evaluated in medium containing glucose or amino acids as the sole carbon source. In the absence of CP, the growth of strains lacking RsaC was similar to the parental strain—wild type and Δ*mntC*Δ*mntH,* respectively—for both glucose- and amino acid-containing media (Fig. 2A and B; Fig. S4A and B). In glucose-containing medium, the addition of CP resulted in Δ*rsaC*Δ*mntC*Δ*mntH* growing worse than Δ*mntC*Δ*mntH* (Fig. 2A and Fig. S4A). A trend towards reduced growth was observed with Δ*rsaC* across multiple CP concentrations but did not reach significance. Ectopic expression of *rsaC* reversed the growth defect of both RsaC mutants (Fig. 2C and Fig. S4C). Together, these results indicate that RsaC is necessary for maximal growth on glucose when *S. aureus* experiences substantial Mn limitation. In amino acid-containing medium including CP, both Δ*rsaC* and Δ*rsaC*Δ*mntC*Δ*mntH* exhibited growth defects relative to their parental strain, with a pattern of complementation observed upon expression of *rsaC* from a plasmid (Fig. 2B and D; Fig. S4B and D). These data indicate that RsaC is also necessary for maximal growth on amino acids when *S. aureus* is Mn-starved. Notably, in amino acid–containing medium, less metal restriction was required to observe growth defects upon loss of RsaC than in glucose-containing medium. Cumulatively, these observations indicate that RsaC contributes to the ability of *S. aureus* to utilize either glucose or amino acids when Mn-starved, thereby supporting the metabolic flexibility of *S. aureus*.

### Concurrent environmental stressors alter the impact of RsaC on staphylococcal fitness

**Fig 3:**
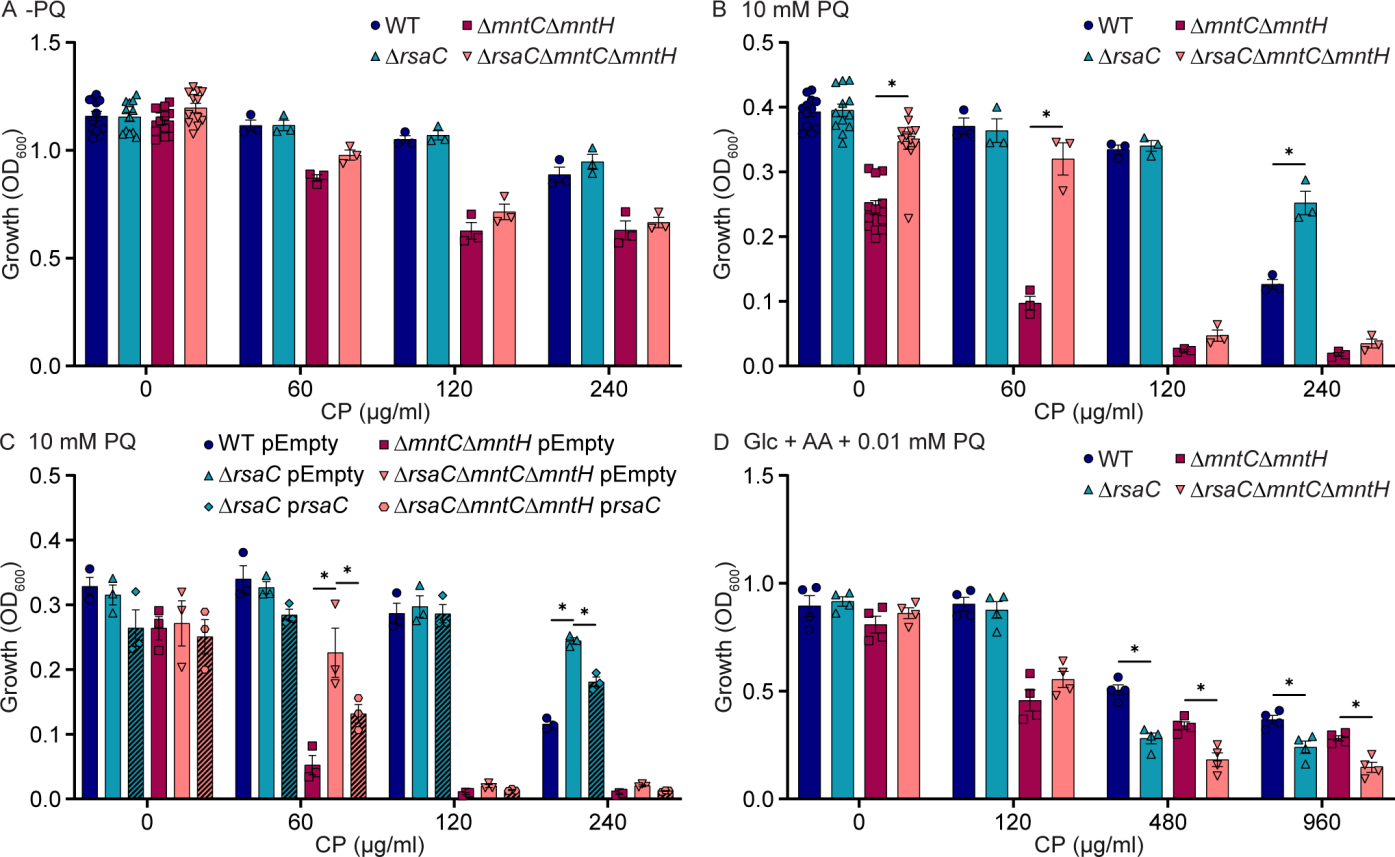
RsaC reduces the ability of S. aureus to survive oxidative stress. (**A–C**) The indicated strains of *S. aureus* were incubated in TSB with CP in the (**A**) absence and (**B, C**) presence of 10 mM PQ, and growth was assessed by measuring optical density after 10 hours. (**C**) As indicated, the strains contain either an empty vector (pEmpty) or an RsaC-expressing plasmid (p*rsaC*). (**D**) The indicated strains were incubated in defined medium containing glucose (Glc) and amino acids (AA) in the presence of CP and 0.01 mM PQ, and growth was assessed by evaluating optical density after 8 hours. *P ≤ 0.05 for the indicated comparison was determined by two-way ANOVA with Šidák’s multiple comparisons test. *n* ≥ 3. Error bars = SEM.

Phagocytes both release CP and elaborate an oxidative burst, forcing pathogens to simultaneously cope with Mn starvation and oxidative stress (10, 15). However, in Mn-restricted environments, the preservation of essential Mn-dependent processes is in conflict with resisting oxidative stress, which also increases the cellular demand for Mn (2, 5). Therefore, the impact of RsaC on staphylococcal growth in the presence of superoxide stress during Mn limitation was evaluated. For these experiments, wild type, Δ*rsaC*, Δ*mntC*Δ*mntH*, and Δ*rsaC*Δ*mntC*Δ*mntH* were grown in TSB, a complex metal-replete medium, in the presence of the superoxide-generating compound paraquat (PQ) and CP. In the absence of CP, wild type and Δ*rsaC* grew similarly both in the presence and absence of 10 mM PQ (Fig. 3A and B; Fig. S5A and B). Differing from the defined medium, in TSB, the loss of RsaC did not impair the growth of *S. aureus* in the presence of CP (Fig. 3A and Fig. S5A). Unexpectedly, in the presence of CP and PQ, Δ*rsaC* grew better than wild type (Fig. 3B and Fig. S5B). In TSB without supplementation, Δ*mntC*Δ*mntH* and Δ*rsaC*Δ*mntC*Δ*mntH* grew similarly to wild type. However, in the presence of 10 mM PQ, consistent with its sensitivity to oxidative stress (5, 24), Δ*mntC*Δ*mntH* grew worse than wild type. The addition of CP exacerbated the growth defect of Δ*mntC*Δ*mntH*. However, the Δ*rsaC*Δ*mntC*Δ*mntH* mutant continued to grow better than Δ*mntC*Δ*mntH* (Fig. 3B and Fig. S5B). Thus, both Δ*rsaC* and Δ*rsaC*Δ*mntC*Δ*mntH* grew better than their parental strain when both CP and PQ were added to the medium. The growth advantage of both strains in the presence of PQ was diminished upon ectopic expression of *rsaC* (Fig. 3C; Fig. S5C) and in the presence of modified CP unable to bind Mn (Δ6His; Fig. S6D) (26).

Aerobic complex media intrinsically and continuously produces reactive oxygen species (29). Therefore, to test the hypothesis that secondary stressors dictate if RsaC benefits or harms the bacterium, the growth of *S. aureus* in the presence of CP and PQ was evaluated using a defined medium containing glucose and amino acids. Across multiple CP concentrations in the presence of PQ, Δ*rsaC* and Δ*rsaC*Δ*mntC*Δ*mntH* grew worse than wild type and Δ*mntC*Δ*mntH*, respectively (Fig. 3D and Fig. S5D). These growth defects were reversed by expression of *rsaC* from a plasmid (Fig. S5E and F). Taken together, these observations support a model in which RsaC has a critical role in enabling *S. aureus* to survive Mn starvation, but its impact is modulated by the environment and presence of other stressors.

### *S. aureus* suppresses SodA expression in response to host-imposed manganese starvation

**Fig 4:**
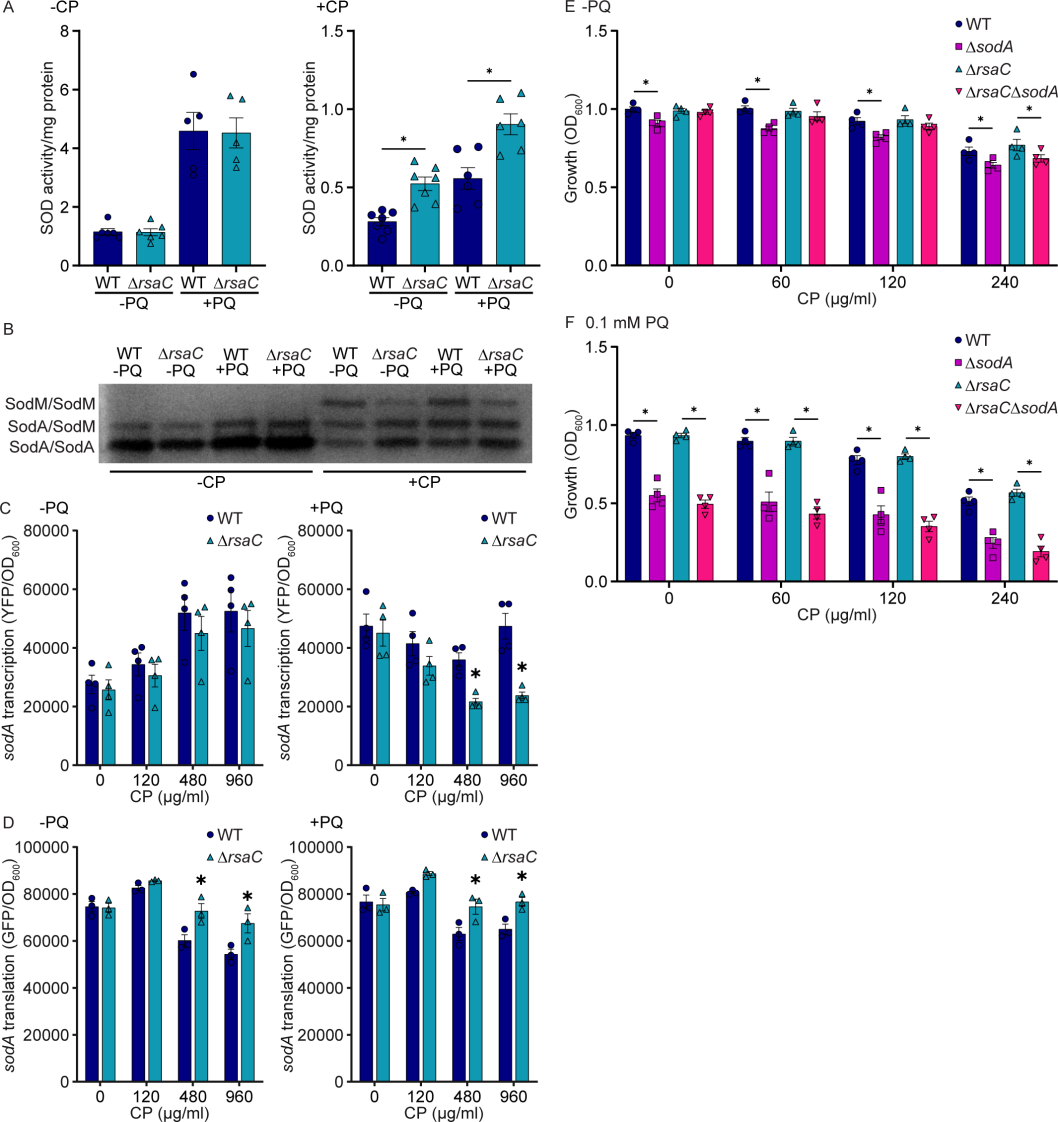
RsaC suppresses SodA expression and activity in the presence of calprotectin. (**A, B**) Cell lysates from the indicated strains were assessed for (**A**) total and (**B**) individual SOD activity following growth in the presence and absence of 240 µg/mL CP and 1 mM PQ. Individual activity was assessed using a zymogen gel, in which the lower band is the SodA homodimer, the middle band is the SodA/SodM heterodimer, and the upper band is the SodM homodimer. Image is a representative of three independent replicates. (**A**) *P ≤ 0.05 of the indicated comparison via unpaired *t* test. (**C, D**) Transcription and translation of *sodA* were assessed utilizing reporter plasmids (pAH5: *sodA*-YFP and pCN52: *sodA*-GFP, respectively) in the indicated strains following 8 hours of growth in the presence and absence of 1 mM PQ and CP. (**C, D**) *P ≤ 0.05 relative to wild type at the same CP concentration via two-way ANOVA with Šidák’s multiple comparison test. (**A–D**) *n* ≥ 3. Error bars = SEM. (**E, F**) The indicated strains of *S. aureus* were incubated in TSB with CP in the (**E**) absence or (**F**) presence of 0.1 mM PQ and growth was assessed by measuring optical density after 8 hours. *P ≤ 0.05 for the indicated comparison was determined by two-way ANOVA with Dunnett’s multiple comparisons test. *n* = 4. Error bars = SEM.

*S. aureus* possesses two superoxide dismutases, the Mn-dependent enzyme SodA and the cambialistic SodM, which can use both Fe and Mn (30, 31). In Mn-replete environments, SodA is the primary source of SOD activity, while SodM is the primary source in Mn-limited environments (32). RsaC suppresses SodA translation when heterologously expressed in Mn-replete media (22). This leads to the hypothesis that loss of RsaC benefits *S. aureus* by increasing SOD activity in the presence of Mn starvation and oxidative stress. To test this, the impact of RsaC on SodA and SodM activity was evaluated in the presence and absence of CP and PQ. In the absence of CP, loss of RsaC did not impact total SOD activity or its distribution (Fig. 4A and B). Consistent with prior observations (2, 32), CP treatment reduced total SOD activity, and especially that associated with SodA, in wild-type bacteria when compared to untreated cells, both in the presence and absence of PQ (Fig. 4A and B). Similarly, the same conditions also reduced the total SOD activity of Δ*rsaC* (Fig. 4A). However, when compared to wild type, total SOD activity in Δ*rsaC* was elevated in the presence of CP, both with and without PQ, with the increase driven by elevated SodA activity (Fig. 4A and B). These observations indicate that RsaC reduces SodA activity, and thus total SOD activity, when *S. aureus* experiences Mn limitation. However, in the presence of CP, with and without the addition of PQ, Δ*rsaC* had reduced SodA activity (Fig. S7), suggesting that SodA activity is impacted by both RsaC regulation and loss of the enzyme’s cofactor. Notably, SodM activity decreased upon loss of RsaC in the presence of CP with and without PQ (Fig. 4B), indicating that it is indirectly positively regulated by RsaC. However, the induction of SodM in wild type is not sufficient to restore the suppressed activity of SodA, resulting in submaximal protection against superoxide.

The reduction in SodA activity, along with the knowledge that RsaC can inhibit the translation of SodA (22), supports the hypothesis that a failure to suppress expression of SodA leads to the improved growth of Δ*rsaC*. As an initial step in testing this hypothesis, the transcription and translation of *sodA* were assessed using reporter fusions. Loss of RsaC led to reduced transcript but increased translation in the presence of CP with and without PQ (Fig. 4C and D). Next, the growth of wild type, Δ*sodA,* Δ*rsaC,* and Δ*rsaC*Δ*sodA* was evaluated in TSB with PQ and CP. Initially, due to the sensitivity of strains lacking SodA, only 0.1 mM PQ was utilized. In the absence of CP and PQ, all the strains had negligible differences compared to wild type (Fig. 4E and Fig. S8A). In Mn-replete medium with PQ, Δ*sodA* and Δ*rsaC*Δ*sodA* grew worse than wild type and Δ*rsaC* (Fig. 4F and Fig. S8B). While the advantage of possessing RsaC was muted in the presence of CP and a lower concentration of PQ, Δ*rsaC*Δ*sodA* grew worse than Δ*rsaC* (Fig. 4F and Fig. S8B). When 1 mM PQ was utilized, a similar pattern was observed (Fig. S9). Given the muted response of Δ*rsaC*, strains lacking both Mn transporters were further examined. In the presence of CP and PQ, Δ*rsaC*Δ*mntC*Δ*mntH* grew better than Δ*mntC*Δ*mntH*. However, the advantage of losing RsaC was lost by Δ*rsaC*Δ*mntC*Δ*mntH*Δ*sodA*, which grew worse than Δ*rsaC*Δ*mntC*Δ*mntH* (Fig. S9B and C). Cumulatively, these observations indicate that via RsaC, *S. aureus* actively suppresses the expression of SodA in response to CP, which in turn inhibits growth in the presence of oxidative stress.

### RsaC suppresses the cellular demand for manganese

**Fig 5:**
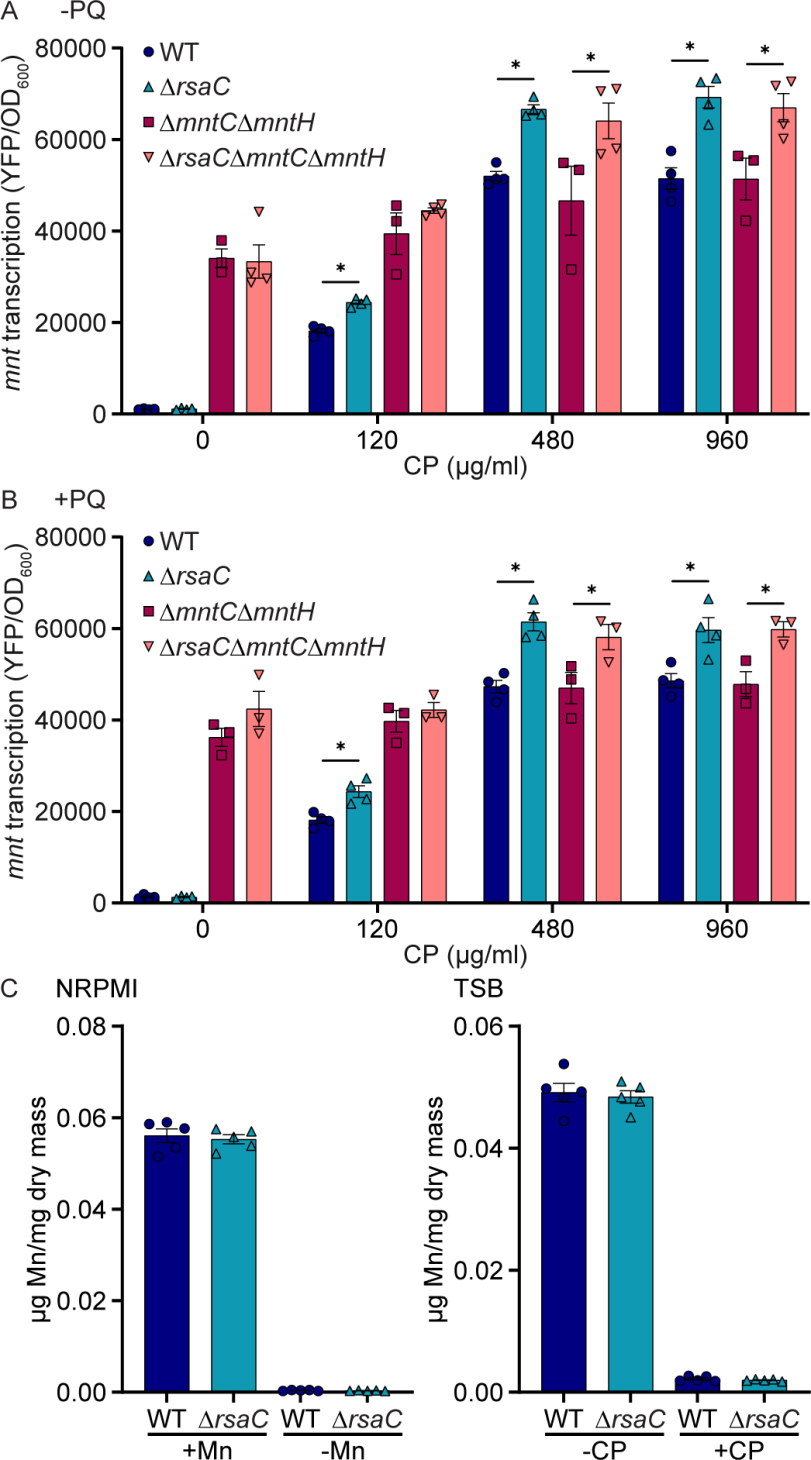
RsaC suppresses the cellular demand for manganese. (**A, B**) The indicated strains of *S. aureus* carrying an *mntABC* transcriptional reporter (P*_mntABC_*-YFP) were grown in TSB with CP in the (**A**) absence or (**B**) presence of 0.01 mM PQ, and expression was assessed by measuring fluorescence after 6 hours. (**A, B**) *P ≤ 0.05 for the indicated comparison was determined by two-way ANOVA with Šidák’s multiple comparisons test. (**C**) Intracellular Mn concentration was measured in *S. aureus* wild type and Δ*rsaC* using ICP-OES following growth to an OD_600_ of ∼0.25 in NRPMI in the presence and absence of Mn or TSB in the presence and absence of 240 µg/mL CP. No comparisons in the same growth condition were significant via unpaired *t* test. (**A–C**) *n* ≥ 3. Error bars = SEM.

The suppression of SodA expression by RsaC in response to CP leads to the hypothesis that this sRNA activates a Mn-sparing response that enables *S. aureus* to survive nutritional immunity. If true, loss of RsaC should increase the extent of Mn limitation experienced by *S. aureus*. To quantify the Mn limitation perceived by *S. aureus*, the expression of *mntABC* was assessed in wild type and Δ*rsaC* using a plasmid-encoded transcriptional reporter following growth in the presence of CP. As expected (24), in both wild type and Δ*rsaC*, *mntABC* was not strongly expressed in the absence of CP (Fig. 5A and B). In the presence of CP, *mntABC* was more highly expressed in Δ*rsaC* than in wild type, consistent with *S. aureus* perceiving more extreme Mn limitation in the absence of RsaC. A similar result was observed upon the addition of PQ (Fig. 5B). Cumulatively, these observations suggest that loss of RsaC increases the staphylococcal demand for Mn.

The expression data are consistent with the hypothesis that RsaC induces a Mn-sparing response. However, they do not exclude the possibility that RsaC impacts Mn transporter expression or Mn accumulation. To address the first possibility, *mntABC* expression was assessed in a Mn transporter-deficient strain Δ*mntC*Δ*mntH* (24). In this strain background, loss of RsaC still led to increased *mntABC* promoter activity (Fig. 5A and B). This indicates that the apparent increase in cellular demand for Mn is not driven by Δ*rsaC* failing to induce the expression of Mn transporters. To address the second possibility, wild type and Δ*rsaC* were grown in metal-defined medium lacking Zn and Fe with and without Mn, or a complex medium containing CP, and cellular metal content was assessed. In both instances, wild type and Δ*rsaC* accumulated similar levels of Mn (Fig. 5C). Cellular accumulation of Zn, Fe, Cu, Mg, Ca, and other metals by both strains had negligible differences (Fig. S10). This analysis indicates that loss of RsaC does not prevent *S. aureus* from acquiring Mn or other metals. In total, these observations support a model in which RsaC controls a Mn-sparing response that suppresses the cellular demand for Mn.

### RsaC is important for *S. aureus* virulence

**Fig 6:**
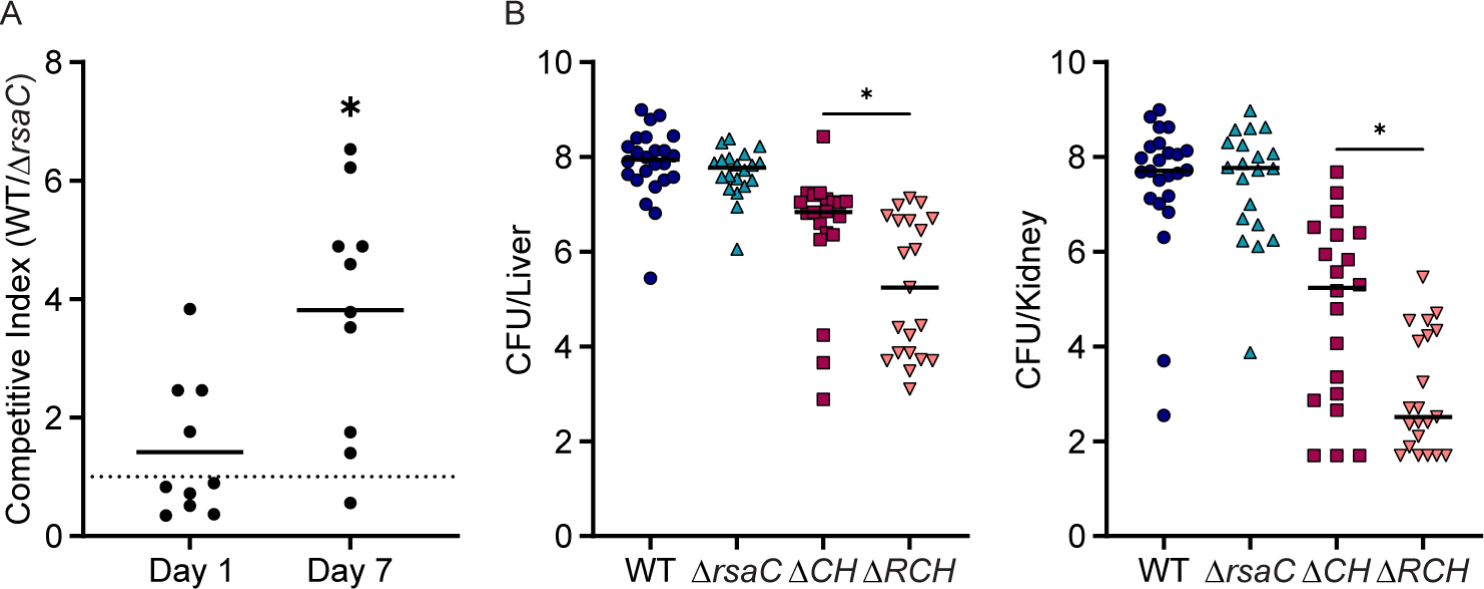
RsaC is important for *S. aureus* virulence. (**A**) Wild-type C57BL/6J mice were subcutaneously infected with an equal ratio of wild type and Δ*rsaC,* and bacterial burdens and competitive index were determined one and seven days post-infection. *P ≤ 0.05 compared to a theoretical mean of 1 via one sample *t*-test. The lines = mean. *n* = 10. (**B**) Wild-type C57BL/6J mice were infected systemically with *S. aureus* wild type, or the indicated strains and bacterial burdens of the indicated organs were enumerated four days post-infection. *P ≤ 0.05 for the indicated comparison was determined by unpaired *t-*test. The lines = median. *n* ≥ 20. Δ*CH* = Δ*mntC*Δ*mntH* Δ*RCH* = Δ*rsaC*Δ*mntC*Δ*mntH*.

RsaC enhances the ability of *S. aureus* to resist Mn starvation but sensitizes the bacterium to oxidative stress. As both threats are encountered during infection, it was unclear whether RsaC would benefit *S. aureus* during infection. *S. aureus* is a significant cause of skin infections. Therefore, a subcutaneous infection model was first used to evaluate the impact of RsaC on infection. Mice were infected with wild type and Δ*rsaC*, and bacterial burdens and competitive index were determined one and seven days post-infection. In this model, Δ*rsaC* was outcompeted by wild-type bacteria after seven days of infection (Fig. 6A). These results indicate that RsaC contributes to the ability of *S. aureus* to survive within the host.

*S. aureus* also causes systemic infections; therefore, the impact of RsaC’s absence was assessed using a systemic infection model (33). Mice were individually infected with either wild type or Δ*rsaC,* and bacterial burdens were assessed four days post-infection. Similar bacterial burdens were recovered for both wild type and Δ*rsaC*, indicating that while the loss of RsaC did not impair the ability of *S. aureus* to cause systemic infection, it also did not overtly benefit the bacterium (Fig. 6B). The current work revealed that the benefit of RsaC is impacted by the extent of Mn limitation experienced by *S. aureus*. Mn levels can vary within the human population, influenced by diet, underlying medical conditions, and genetics, and this variation alters the outcome of infection (34–37). This leads to the hypothesis that, during systemic infection, the benefit of possessing RsaC is influenced by the host’s Mn status. To test this hypothesis without disrupting other host processes, a Δ*mntC*Δ*mntH* mutant lacking the two Mn transporters was utilized, reducing the ability of *S. aureus* to obtain Mn. Mice were infected with Δ*mntC*Δ*mntH* and Δ*rsaC*Δ*mntC*Δ*mntH*. In both the liver and kidney, fewer colony-forming units were recovered following infection with Δ*rsaC*Δ*mntC*Δ*mntH* than with Δ*mntC*Δ*mntH* (Fig. 6B). These results reveal that RsaC contributes to staphylococcal infection, with its impact influenced by the extent of Mn starvation experienced by *S. aureus* within a tissue.

## DISCUSSION

**Fig 7:**
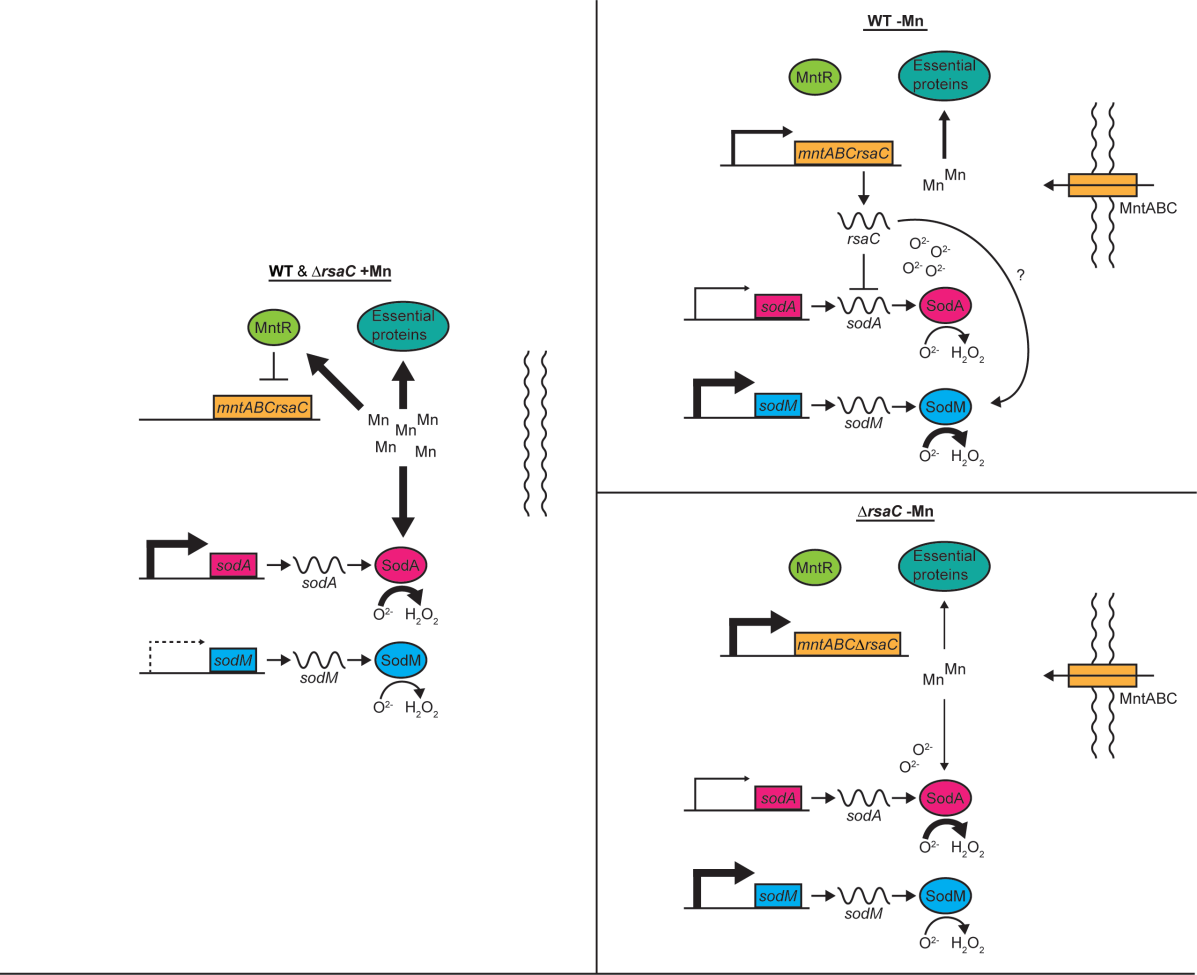
RsaC triggers a Mn-sparing response. In Mn-replete media, the transcriptional regulator MntR suppresses the *mntABCrsaC* operon. In this Mn-replete environment, Mn-utilizing proteins, including SodA, are robustly expressed and active. In the presence of CP or in other Mn-limited environments, expression of the *mntABCrsaC* operon is no longer repressed by MntR, leading to the production of RsaC. RsaC suppresses the translation of the Mn-dependent SodA*,* and by an unknown mechanism induces the expression of the Fe-utilizing SodM. The suppression of SodA expression and activity sensitizes *S. aureus* to superoxide stress but reduces the cellular demand for Mn, allowing this metal to be allocated to alternative Mn-dependent processes that are essential for growth. Line thickness indicates strength of the given process. ? indicates an unknown mechanism.

Bacteria must adapt to the environment, including in the presence of stressors, which impose conflicting demands on the cell. Thus, to survive, bacteria must coordinate and balance their responses, elaborating a response that is suboptimal for one stressor to ensure viability in the presence of a second stressor (1, 2). The current investigation establishes that RsaC mediates a Mn-sparing response, which is important for *S. aureus* to navigate changes in Mn availability (Fig. 7). However, the impact of RsaC on staphylococcal survival is dependent on the environment, with the loss of this sRNA both promoting and suppressing survival. In the absence of other stressors, RsaC promotes *S. aureus* growth under Mn-limiting conditions as it reduces the cellular demand for Mn. However, when oxidative stress is present, loss of RsaC benefits *S. aureus,* due to the suppression of SodA by this sRNA. This dichotomy is revealed during infection, where the importance of RsaC to pathogenesis depends on the extent of Mn starvation experienced by *S. aureus* within a tissue. Beyond establishing a critical role for RsaC in enabling *S. aureus* to overcome nutritional immunity, this study reveals a critical role for this sRNA in triggering a Mn-sparing response and balancing mutually exclusive stress responses, limiting Mn utilization while resisting oxidative stress.

Small RNA-driven responses to Fe scarcity are well established, but whether they contribute to surviving the absence of other metals was unknown (18). The contribution of RsaC to overcoming host-imposed Mn starvation and suppressing the cellular demand for Mn reveals that sRNAs can limit the use of non-Fe metals and promote survival in metal-depleted environments. This suggests that sRNAs have a larger role in controlling metal homeostasis and the response to inorganic nutrient availability than previously thought. In *Streptomyces coelicolor*, a nickel-responsive sRNA s-SodF suppresses the Ni-SOD upon Ni limitation (38). Similarly, *Helicobacter pylori* possesses NikS, whose expression is controlled by Ni availability (39). However, it is unknown if either of these sRNAs activate a Ni-sparing response that promotes survival. Beyond metals, microbes elaborate sparing responses in response to the absence of other critical nutrients (40). A prominent example is the bacterial response to phosphate limitation, which frequently involves reduced production of phosphate-rich teichoic acids, enhanced production of sulfur-rich teichuronic acids, and the induction of sRNA, among other adaptations (41, 42). This parallels changes mediated by sRNAs in response to metal availability, raising the possibility that sRNAs might more broadly have critical roles in coordinating bacterial responses to inorganic nutrient availability.

The need to balance competing imperatives is not restricted to the conflicting need to simultaneously maintain SOD activity and other Mn-dependent processes (43–45). For instance, glucose consumption is required for nitric oxide resistance during infection, but glycolysis increases the cellular demand for Mn in the already Mn-limited infection environment (1, 27). Pathogens, including *Mycobacterium tuberculosis*, *Salmonella*, and *Pseudomonas aeruginosa*, reduce their growth rate and pathogenicity during infection to enable antibiotic resistance (46–50). These compromises arise from mutations that promote resistance but hamper growth (51–54). Leveraging regulatory circuits rather than mutations enables bacteria to temporarily, rather than permanently, occupy a suboptimal state. Understanding how pathogens cope with individual stressors is important for revealing their potential and ideal response. However, the need for RsaC during infection, despite its role in dampening SOD activity, emphasizes that within a native environment, microbes must deviate from their ideal responses to survive. Failing to consider the totality of stressors in an environment can lead to a false picture of the paths available to microbes and falsely suggest that microbes possess redundant capacity. Examples of false redundancy include the metal-independent glycolytic enzymes in *S. aureus* and *S. enterica* Typhimurium, which are critical for maintaining glycolytic flux and infection, but only when Mn is restricted. Similarly, in *S. aureus*, SodM is critical when Mn is restricted, while in *E. coli,* SodA becomes important when Fe is restricted, but only in the presence of superoxide stress (32, 55–57).

The active suppression of SodA by RsaC in *S. aureus*, and of Fe-dependent SODs by RyhB-like sRNAs (22, 58), highlights the critical need to control the expression of SODs, which bind their cofactor irreversibly, when metal availability is restricted. However, the molecular details of how the SODs of *S. aureus* and other pathogens are regulated are limited, with investigations frequently focusing solely on the impact of oxidative stress (30, 32, 59). This is despite evidence that pathogens simultaneously experience metal starvation and oxidative stress during infection, as demonstrated by the release of both ROS and metal-sequestering proteins, including CP and lactoferrin, by neutrophils (2, 10, 31, 32, 60). While RsaC dampens SodA expression to suppress the cellular demand for Mn, it enhances the expression of SodM, likely via an indirect mechanism (22). This suggests that the expression of SodM, and its ability to utilize Fe, has been selected for as a defense against host-imposed Mn starvation. While the full extent of RsaC’s targets is unknown, its contribution to metabolic flexibility by enabling the utilization of both glucose and amino acids suggests that the regulatory network of this sRNA and its contribution to resisting Mn starvation extends beyond the SODs and thus broadly contributes to overcoming nutritional immunity.

The current study reveals that sRNAs have a greater role in coordinating the bacterial response to metal availability during infection than previously appreciated. RsaC sits at a nexus balancing competing imperatives, resisting oxidative stress and surviving Mn starvation, and contributes to the metabolic flexibility known to be important for *S. aureus* virulence. As a result, its importance is dictated by nutrient content of the environment and presumptively the extent of oxidative stress experienced by *S. aureus*. Notably, both of these factors can vary between individuals and geographical regions, driven by genetics, diet, and underlying health status (35). There is also variation in RsaC across staphylococcal lineages (22). Thus, further investigation of its function in staphylococcal physiology will advance our understanding of how bacteria adapt to the presence of multiple stressors that impose conflicting demands on the cell, and the molecular details of how these conflicts are resolved.

## MATERIALS AND METHODS

### Bacterial strain and plasmid construction

Bacterial strains were stored in brain-heart infusion (BHI) supplemented with 30% glycerol at −80°C. *S. aureus* strains were routinely cultured in 5 mL TSB in a 15 mL conical tube and on tryptic soy agar (TSA) plates. *Escherichia coli* strains were routinely cultured in 5 mL Luria-Bertani broth (LB) in round-bottom glass culture tubes and on Luria-Bertani agar (LBA) plates. Chloramphenicol, ampicillin, erythromycin, kanamycin, and tetracycline were used at 10 µg/mL, 100 µg/mL, 10 µg/mL, 250 µg/mL, and 1 µg/mL, respectively.

The strains used in this study are listed in Table S1. Staphylococcal mutants were generated using established protocols for allelic replacement and phage transduction (61). All plasmids were sequenced prior to use, and the hemolytic activity of mutants was confirmed by plating on blood agar. To construct Δ*rsaC*, a construct for deleting *rsaC* was created in pKORI by amplifying the 5′ and 3′ flanking regions using the primers indicated in Table S2, and Gibson assembly. To create Δ*rsaC*Δ*mntC*Δ*mntH,* a construct for deleting *mntC* and *rsaC* was created in pKORI by amplifying the 5′ and 3′ flanking regions using the primers indicated in Table S2 and Gibson Assembly. To eliminate *mntH*, an *mntH::erm* allele from the Nebraska Transposon Mutant Library (NTML) (62, 63) was transduced into Δ*rsaC*Δ*mntC*. To create Δ*mntC*Δ*mntH*Δ*sodA::tet* and Δ*rsaC*Δ*mntC*Δ*mntH::erm*Δ*sodA::tet*, a *sodA::tet* allele (31) was transduced into Δ*mntC*Δ*mntH* and Δ*rsaC*Δ*mntC*Δ*mntH::erm,* respectively.

The plasmids used in this study are listed in Table S3 and were introduced into the recipient strains via Phi85 transduction or electroporation. To construct the SodA translational reporter, the full-length 5′UTR (64) of *sodA* and 11 codons past the start codon were amplified using the primers listed in Table S2 and fused to the pHELP promoter using the primers listed in Table S2. This construct was introduced into pCN52, controlling GFP expression. For complementation studies, *rsaC* was cloned into the pOS1 vector under the control of the *lgt* promoter using the indicated primers in Table S2.

### Northern blot analysis

*S. aureus* HG001 (65) wild-type cells were grown in NRPMI medium supplemented with 1 mM MgCl_2_, 100 µM CaCl_2_, ±25 µM MnCl_2_, ±25 µM ZnCl_2_, and ±1 µM FeSO_4_ and collected at OD_600_ = 1. After centrifugation, pellets were resuspended in RNA Pro Solution (FastRNA Pro Blue Kit; MP Biomedicals) and lysed with a sample disruption instrument (FastPrep; MP Biomedicals). Total RNA was extracted according to the manufacturer’s instructions. The RNA samples were then run on a 1% agarose gel containing 25 mM guanidinium thiocyanate (Sigma-Aldrich). After electrophoresis, RNA was transferred onto a Hybond N+ nitrocellulose membrane (GE Healthcare Life Sciences) by capillarity. The membrane was then hybridized with DIG-labeled probes specific to RsaC sRNA and 5S rRNA (loading control), produced using the DIG RNA Labeling Kit (Roche). For detection, anti-digoxigenin-AP Fab fragments and CDP-Star (Roche) were used. Membranes were exposed to X-ray films (Fuji) and developed in an Optimax X-ray film processor. The results are representative of two independent experiments. RsaC DIG probe was amplified with TAATACGACTCACTATAGGGAAAAGCTTTATGTGG (forward) and AAAATAGCCACACTCATATG (reverse), and 5S DIG probe was amplified using TAATACGACTCACTATAGGGGATTTGTCATTTGCCTGGC (forward) and GTAAGTTATTTTGTCTGGTGGCTATAGC (reverse).

### Growth assays

CP growth assays were performed, as described previously, with modifications (2, 26). Bacteria were grown overnight for 16 hours in 5 mL of the specified growth medium in a 15 mL conical tube on a roller drum at 37°C. Following this, the bacterial cultures were used directly or diluted 1:10 into TSB as indicated. The undiluted or diluted cultures were then used to inoculate 96-well round-bottom plates containing 100  µL of growth medium and various concentrations of CP. The assay medium consisted of 38% medium (TSB or carbon source defined medium) and 62% CP buffer (20  mM Tris [pH 7.5], 100  mM NaCl, 1  mM CaCl_2_). Calprotectin, a Cys to Ser mutant that retains wild-type metal binding and activity, was purified as previously described (2, 66). To generate Δ6His CP, four histidine ligands were changed to asparagines, and Δ3HisΔAsp CP had three histidines changed to asparagines and the aspartic acid changed to serine (26). The bacteria were incubated with orbital shaking (180 rpm) at 37°C, and growth was measured by assessing the optical density (OD_600_) every one to two hours. Prior to measuring the optical density, the plate was gently vortexed to resuspend the bacteria. As needed, paraquat was added to the growth medium to induce oxidative stress. For assays using a defined carbon source, the basal medium was prepared, as previously described (5) (Table S4). Glucose and casamino acids were added to the concentrated defined media at 1.3% and 6.5%, respectively. The pH of the defined medium was adjusted to 6.8 using 5 M NaOH after the addition of the carbon source, and then metals were brought to a final concentration of 6  mM MgSO_4_, 1  µM FeCl_2_, 1  µM MnCl_2_, and 1  µM ZnSO_4_. For experiments using NRPMI, RPMI supplemented with 1% casamino acids was chelex-treated, as previously described, and supplemented with 1 mM MgCl_2_ and 100 µM CaCl_2_, and 1  µM FeCl_2_, 1  µM MnCl_2_, and 1  µM ZnSO_4_, as indicated (24). The bacteria were grown overnight as described for the CP assays, with the exception that NRPMI lacking transition metals was used instead of TSB. The overnight culture was diluted 1:100 into a round-bottom plate containing 100 µL NRPMI with the indicated metals, and growth was then assessed as for the CP growth assays.

### Expression assays

For the transcriptional and translational reporter assays, bacteria were grown overnight as for the CP assays with minor modifications. For translational reporter assays, the bacteria were precultured in TSB medium and diluted 1:100 into the 96-well plate. For the transcriptional reporter assays, the bacteria were precultured in NRPMI medium with 1% casamino acids, 1  mM MgCl_2_, 100  µM CaCl_2_, and 1  µM FeCl_2_ and were diluted 1:10 into fresh medium and then 1:100 into the 96-well plate. Expression was measured by assessing excitation (505 nm for transcriptional and 485 nm for translational) and emission (535 nm for transcriptional and 528 nm for translational).

### SOD activity assays

Bacteria were grown in the same conditions used for the CP growth assays in TSB, in the presence and absence of 240 µg/mL CP and 1 mM PQ. Once the bacteria reached exponential phase (OD_600_ = ∼0.25), they then were pelleted and resuspended in 0.5  mM KPO_4_ at pH 7.8 with 0.1  mM EDTA (67). The bacteria were then lysed by mechanical disruption, followed by centrifugation to remove insoluble material (31). The protein concentration of the cell lysate was determined using a bicinchoninic acid (BCA) assay kit, while SOD activity was assessed via gel or liquid-based activity assay (2, 68). To visualize individual SOD activity, lysates were resolved on a 10% native polyacrylamide gel, with equivalent amounts of protein being added to each lane. The gels were then incubated in buffer containing 0.05 M KPO_4_ at pH 7.8 with 1  mM EDTA, 0.25  mM nitro blue tetrazolium chloride, and 0.05  mM riboflavin and exposed to fluorescent light, as previously described (68). Gels were imaged using an iBright FL1500 Imaging System. Total SOD activity in cell lysates was assessed using the SOD Activity Assay Kit (Sigma-Aldrich), as per the manufacturer’s instructions, and then normalized to protein content.

### Elemental analyses of *S. aureus* cells

To prepare cells for elemental analyses, wild-type and Δ*rsaC* bacteria were grown using either the same culturing parameters described for TSB-based CP growth assays, with and without the addition of 240 µg/mL CP, or the culturing parameters described for NRPMI-based growth assays, with and without the addition of 1 µM MnCl_2_. Once the bacteria reached exponential phase (OD_600_ = ∼0.25), they were centrifuged and washed two times with 0.1 M EDTA and then two times with MilliQ water. Following the final centrifugation, the cells were resuspended in 1 mL MilliQ water, placed into a pre-weighed tube, and a portion of bacteria was collected for CFU determination. The remaining bacteria were pelleted, and supernatant was removed. Pellets were desiccated overnight by heating at 96°C and then weighed to determine the mass of the dried pellet. Each cell pellet was digested by addition of 0.5 mL concentrated nitric acid (65%, Merck) and heated at 70°C for 16 hours until the biomass was completely digested. The acid digests were diluted tenfold into a dilute (1%) nitric acid solution, prepared in Milli-Q water and containing 50 µg/L Ir as internal standard. Matrix-matched standard solutions containing Mn, Fe, Cu, Zn, Ca, and Mg were prepared in an identical manner for generation of a calibration curve. All standard and sample solutions were analyzed by inductively coupled plasma optical emission spectrometry (ICP-OES) on a Thermo iCAP PRO instrument (RF power 1,250 W, with nebulizer gas flow 0.5 L/min, auxiliary gas flow 0.5 L/min, and cool gas flow 13.5 L/min argon). Elemental concentrations in each sample were calculated by comparison with the standard curve with Qtegra ISDS Software (Thermo) and standardized to biomass according to OD measurements.

### Animal experiments

Ten-week-old mice were used for all of the infection experiments. For both the systemic and subcutaneous infection models, bacteria were grown overnight in 5 mL TSB in a 15 mL conical tube on a roller drum at 37°C. For subcutaneous infections, the bacteria were then diluted 1:50 into fresh 10 mL TSB in 50 mL conical tubes and grown to early log phase (three hours) in a shaking incubator at 180 rpm 37°C. For the systemic infections, overnight cultures were diluted 1:100 into fresh 5 mL of TSB in 15 mL conical tubes and grown to early log phase (three hours) on a roller drum at 37°C. The bacteria were then centrifuged at 4,000 rpm for 10 minutes at 4°C and resuspended in phosphate-buffered saline (PBS). For the subcutaneous infections, the bacteria were resuspended to 1 × 10^9^ CFU/mL, with the two competing strains mixed in a 1:1 ratio. Prior to the infection, the flanks of the mice were shaved and treated with Nair. The mice were then subcutaneously injected with 50 µL (5 × 10^7^ CFU). At the time of sacrifice, infected tissues were harvested and homogenized in 2 mL PBS. Bacterial burdens were enumerated on TSA plates containing kanamycin, tetracycline, or trimethoprim. For systemic infections, the bacteria were resuspended into 1 × 10^8^ CFU/mL, and 100 µL (1 × 10^7^ CFU) was retro-orbitally injected. After four days of infection, the mice were sacrificed, and the livers, kidneys, and hearts were harvested. The livers were homogenized in 5 mL PBS, and the kidneys and hearts were homogenized in 500 µL PBS. CFU were enumerated by plating on TSA plates.

## SUPPLEMENTAL MATERIAL

Supplemental Figures and Tables mbio.01439-25-s0001.docx Figures S1–S10 and Tables S1–S4.

ASM does not own the copyrights to Supplemental Material that may be linked to, or accessed through, an article. The authors have granted ASM a non-exclusive, world-wide license to publish the Supplemental Material files. Please contact the corresponding author directly for reuse.
