# Supplementary material for: A manganese-sparing response balances competing cellular demands to enable Staphylococcus aureus infection
Source: mBio. 2025 Aug 18;16(9):e01439-25. doi: 10.1128/mbio.01439-25 (PMC12421985; doi:10.1128/mbio.01439-25)
Supplement: Supplemental Figures and Tables — Figures S1–S10 and Tables S1–S4. [file mbio.01439-25-s0001.docx]

**Supplemental Figures**


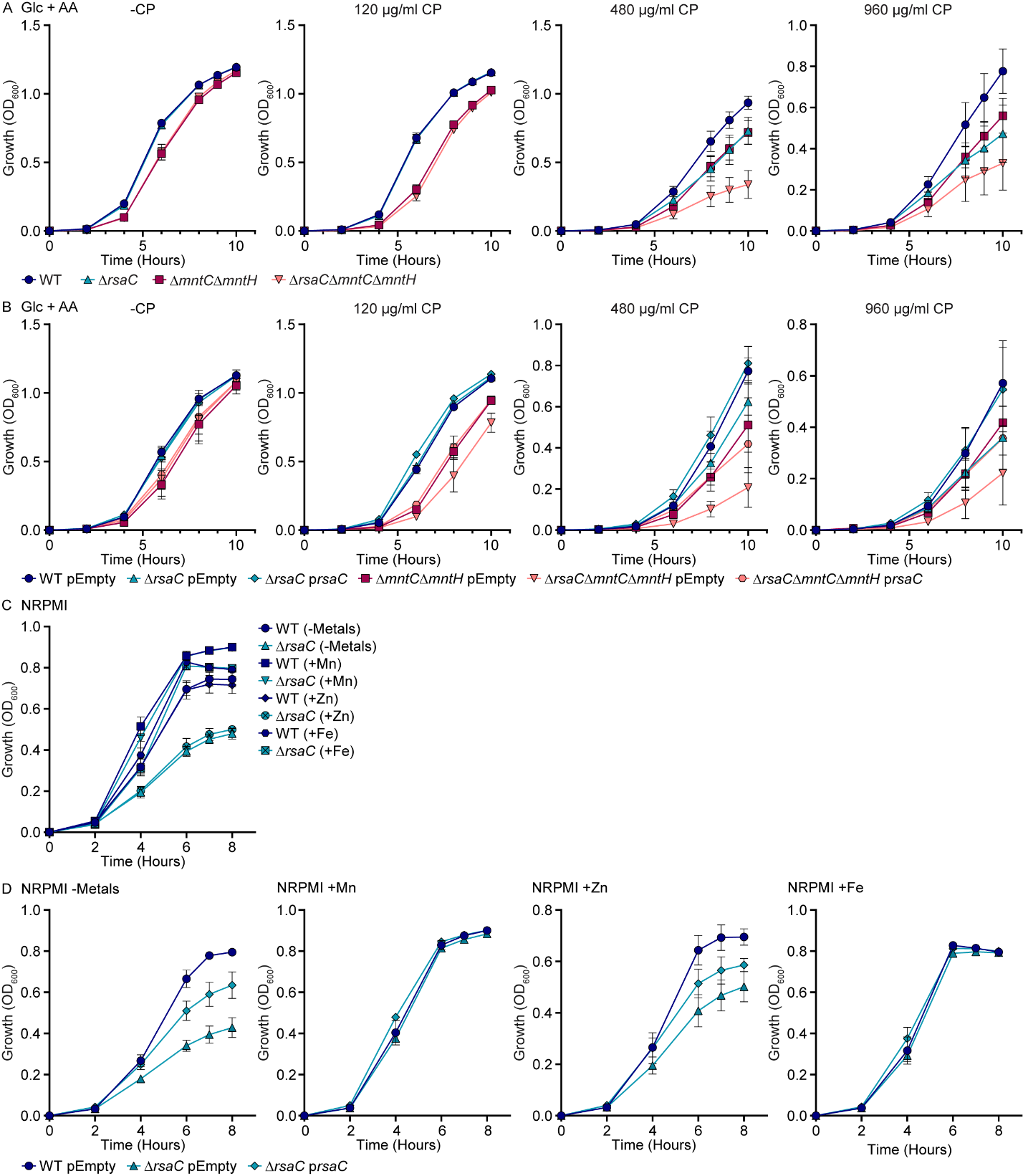


**Supplemental Figure 1. Growth curves in defined medium across various CP concentrations or NRPMI containing various metals.**

(A, B) The indicated strains of *S. aureus* were incubated in defined medium containing glucose (Glc) and amino acids (AA) as carbon sources in the presence of CP and growth was determined by assessing optical density over 10 h. (C, D) The indicated strains of *S. aureus* were incubated in metal limited NRPMI supplemented with 1 µM MnCl_2_, ZnSO_4_, or FeSO_4_ as indicated and growth was assessed over 8 h by measuring optical density. (B, D) As indicated, the strains contain either an empty vector (pEmpty) or an RsaC-expressing plasmid (p*rsaC*). (A-D) n $\geq$ 3. Error bars = SD.


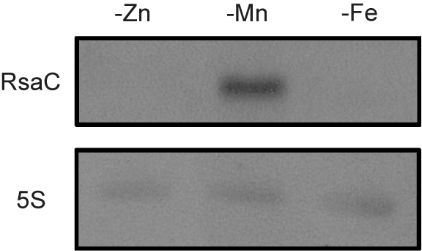


**Supplemental Figure 2. RsaC is produced in response to Mn limitation.**

Northern blot analysis of *rsaC* transcript in wild type *S. aureus* following growth in metal limited NRPMI with and without the addition of 25 µM MnCl_2_, ZnSO_4_, and FeSO_4_ as indicated. Image is a representative of 3 independent replicates.


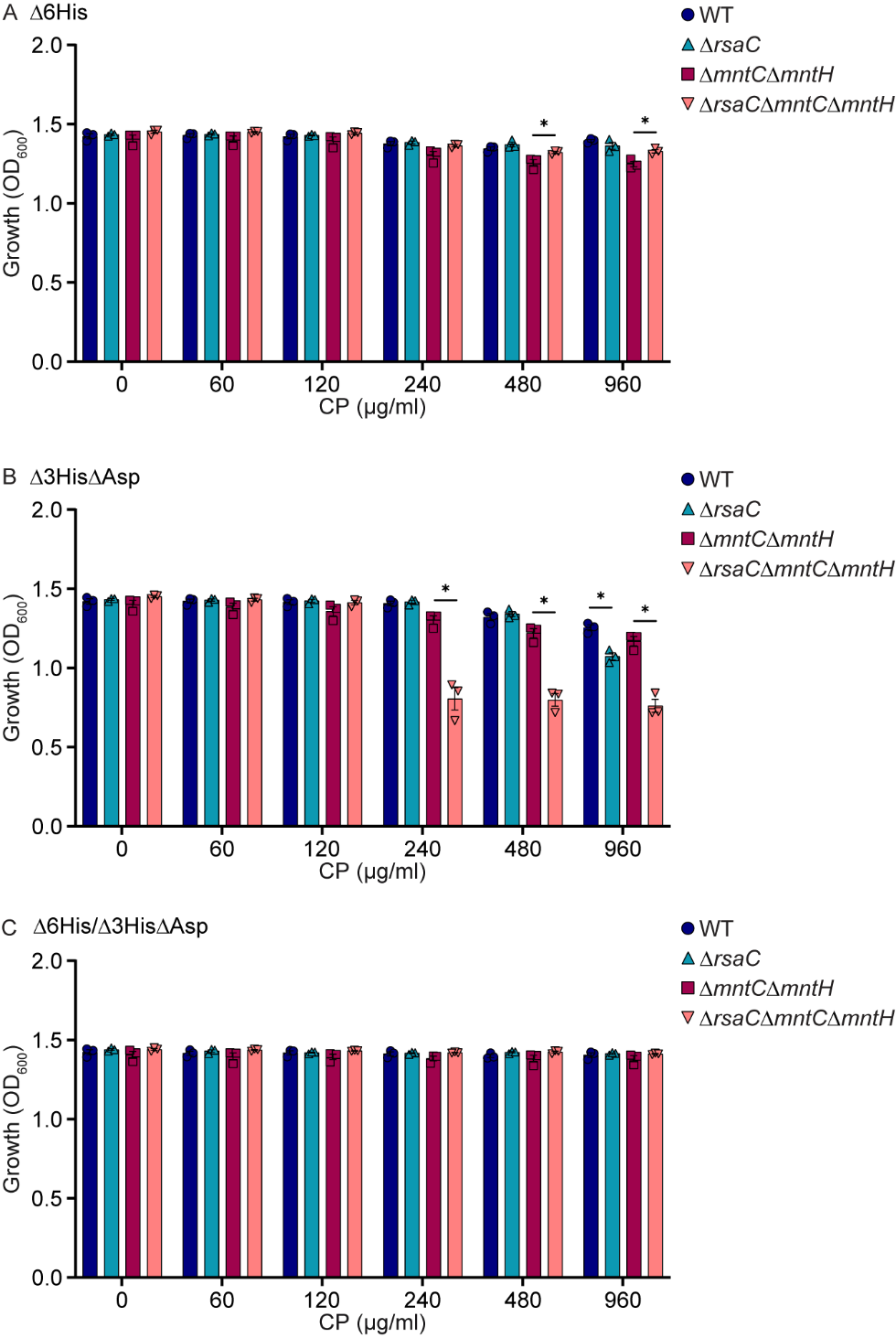


**Supplemental Figure 3. Bacterial growth in defined medium in the presence CP variants with altered metal binding properties.**

The indicated strains of *S. aureus* were incubated in defined medium containing glucose and amino acids as carbon sources in the presence of metal binding variants of CP. Δ6His CP retains the ability to bind Zn, Δ3HisΔAsp CP retains the ability to bind Mn and Zn, and Δ6His/Δ3HisΔAsp cannot bind either metal. The total metal binding capacity of the single site mutants is half that of WT CP. Growth was determined by assessing optical density after 10 h. * = p $\leq$ 0.05 of the indicated comparison via two-way ANOVA with Šidák’s multiple comparisons test. n = 3. Error bars = SEM.


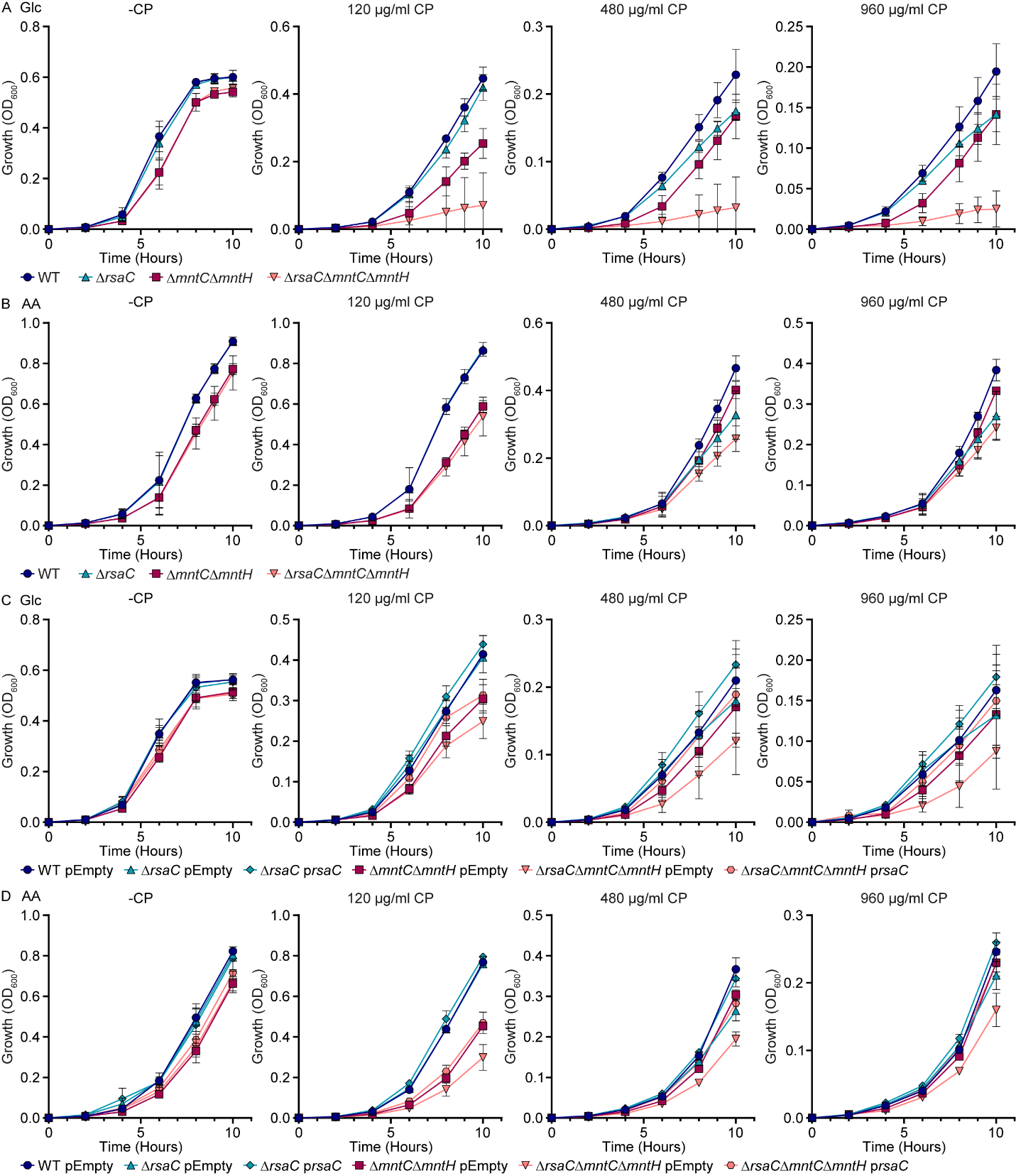


**Supplemental Figure 4. Growth curves in defined medium across various CP concentrations.**

The indicated strains of *S. aureus* were incubated in defined medium containing either (A, C) glucose (Glc) or (B, D) amino acids (AA) as the sole carbon source in the presence of CP and growth was assessed by evaluating optical density over 10 h. (C, D) As indicated, the strains contain either an empty vector (pEmpty) or an RsaC-expressing plasmid (p*rsaC*). (A-D) n $\geq$ 3. Error bars = SD.


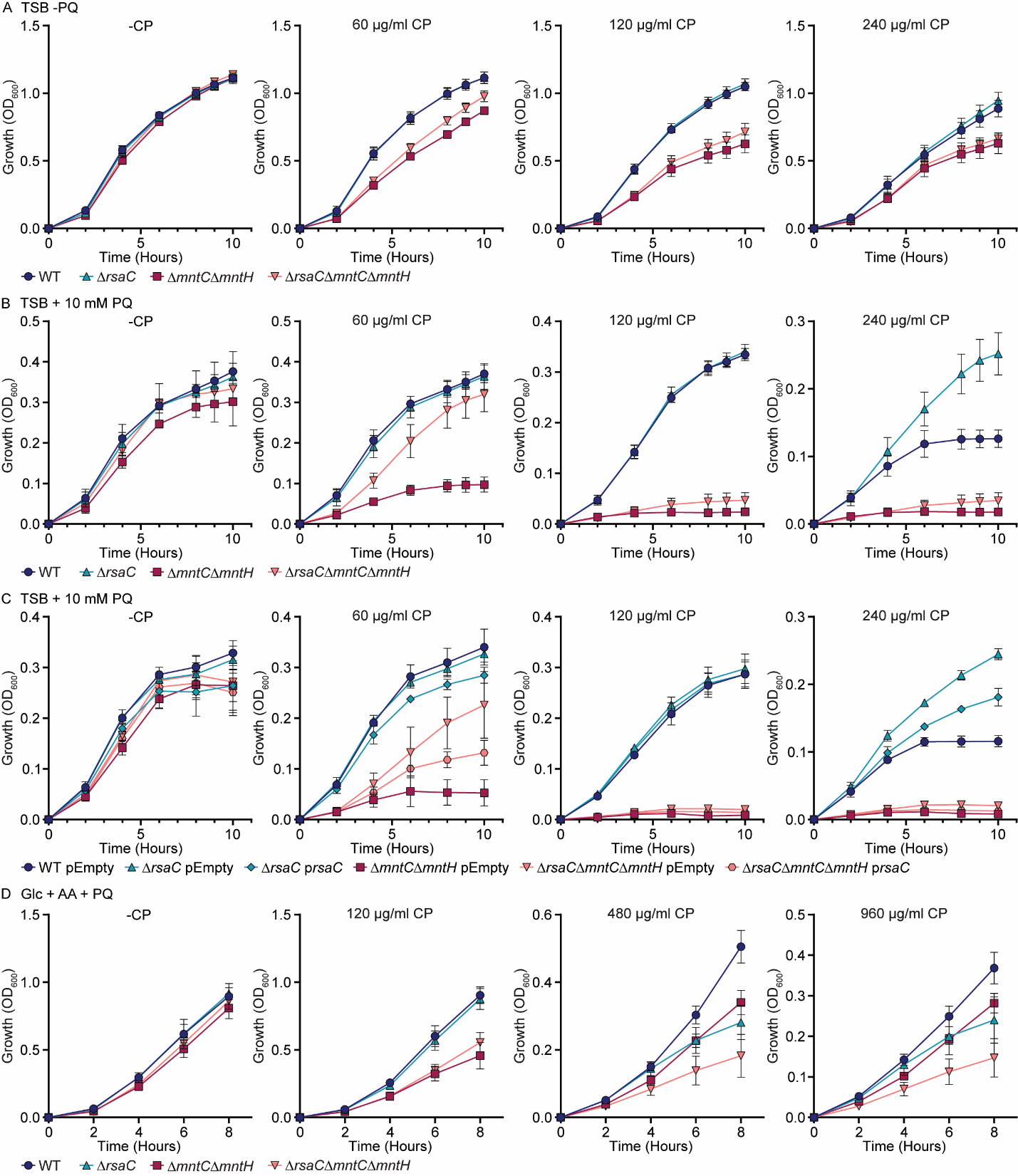


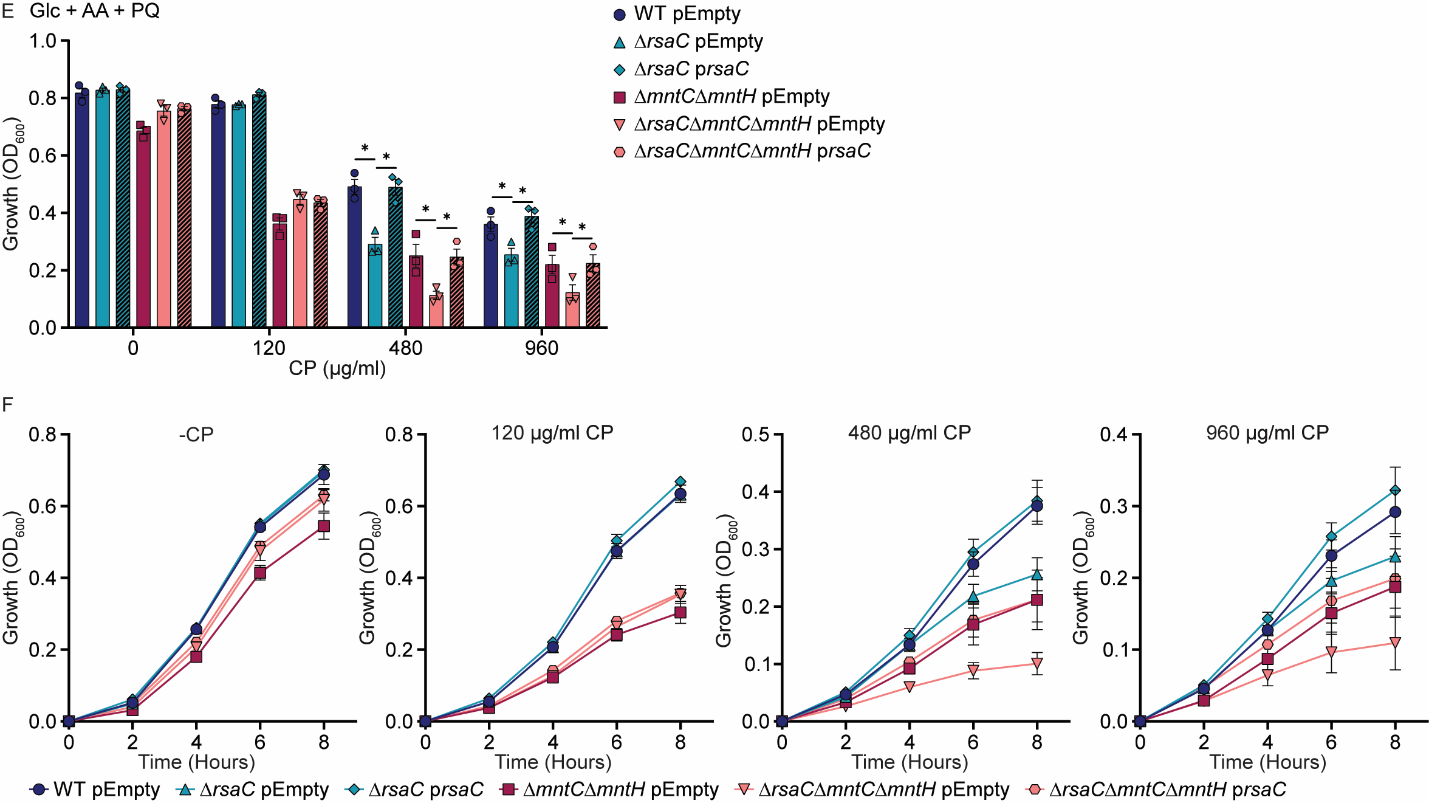


**Supplemental Figure 5.** **Bacterial growth in TSB or defined medium with and without PQ across various CP concentrations.**

(A-C) The indicated strains of *S. aureus* were incubated in TSB with CP in the (A) absence and (B, C) presence of 10 mM PQ and growth was assessed by measuring optical density over 10 h. (D-F) The indicated strains of *S. aureus* were incubated in defined medium containing glucose (Glc) and amino acids (AA) in the presence of CP and 0.01 mM PQ and growth was assessed by evaluating optical density over 8 h. (C, E, F) As indicated, the strains contain either an empty vector (pEmpty) or an RsaC-expressing plasmid (p*rsaC*). (E) * = p $\leq$ 0.05 of the indicated comparison via two-way ANOVA with Šidák’s multiple comparisons test. (A-F) n $\geq$ 3. (A-D, F) Error bars = SD. (E) Error bars = SEM.


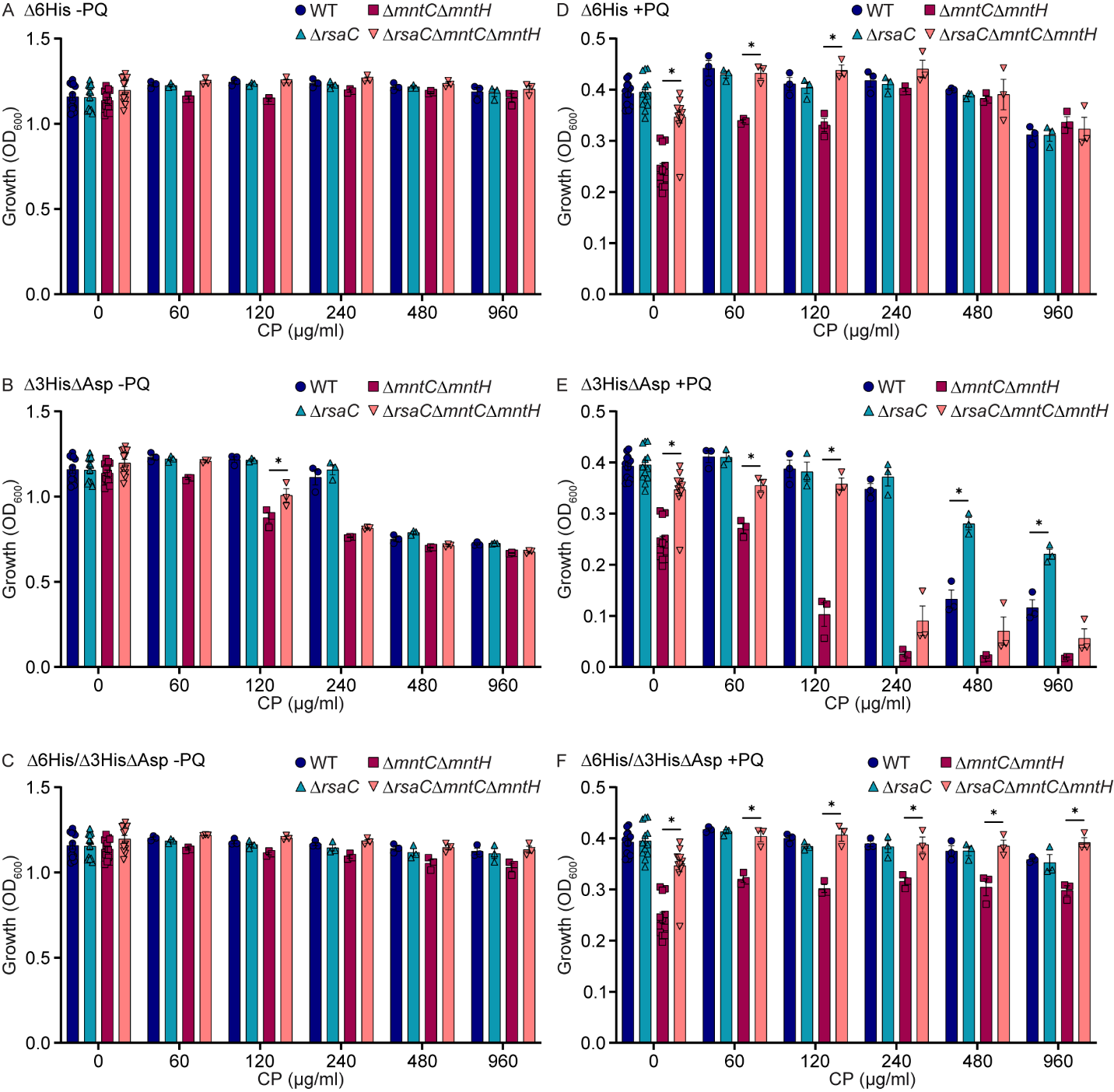


**Supplemental Figure 6.**  **Bacterial growth in TSB with and without PQ in the presence of metal binding CP variants.**

The indicated strains of *S. aureus* were incubated in TSB with modified forms of CP in the (A-C) absence and (D-F) presence of 10 mM PQ. Δ6His CP retains the ability to bind Zn, Δ3HisΔAsp CP retains the ability to bind Mn and Zn, and Δ6His/Δ3HisΔAsp CP cannot bind either metal. Growth was determined by assessing optical density after 10 h. * = p $\leq$ 0.05 of the indicated comparison via two-way ANOVA with Šidák’s multiple comparisons test. n $\geq$ 3. Error bars = SEM. Data from Figure 3A & B growth without CP (without (A) and with (B) PQ) are included in each panel for comparison.


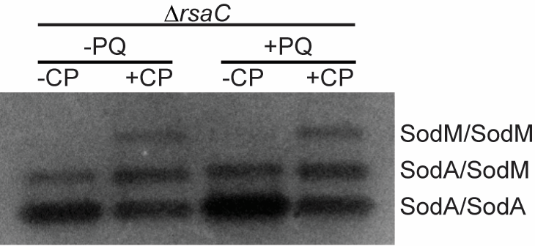


**Supplemental Figure 7.** **SodA activity is controlled by RsaC regulation and cofactor availability.**

Following the growth of Δ*rsaC* the presence and absence of 240 µg/mL CP and 1 mM PQ, cell lysates were assessed for SodA and SodM activity by zymogen gel. The lower band indicates SodA homodimer, the middle band indicates SodA/SodM heterodimer, and the upper band indicates SodM homodimer. The samples are the same as those in Fig. 4B, re-run in a different order to enable visual comparisons. Image is a representative of 3 independent replicates.


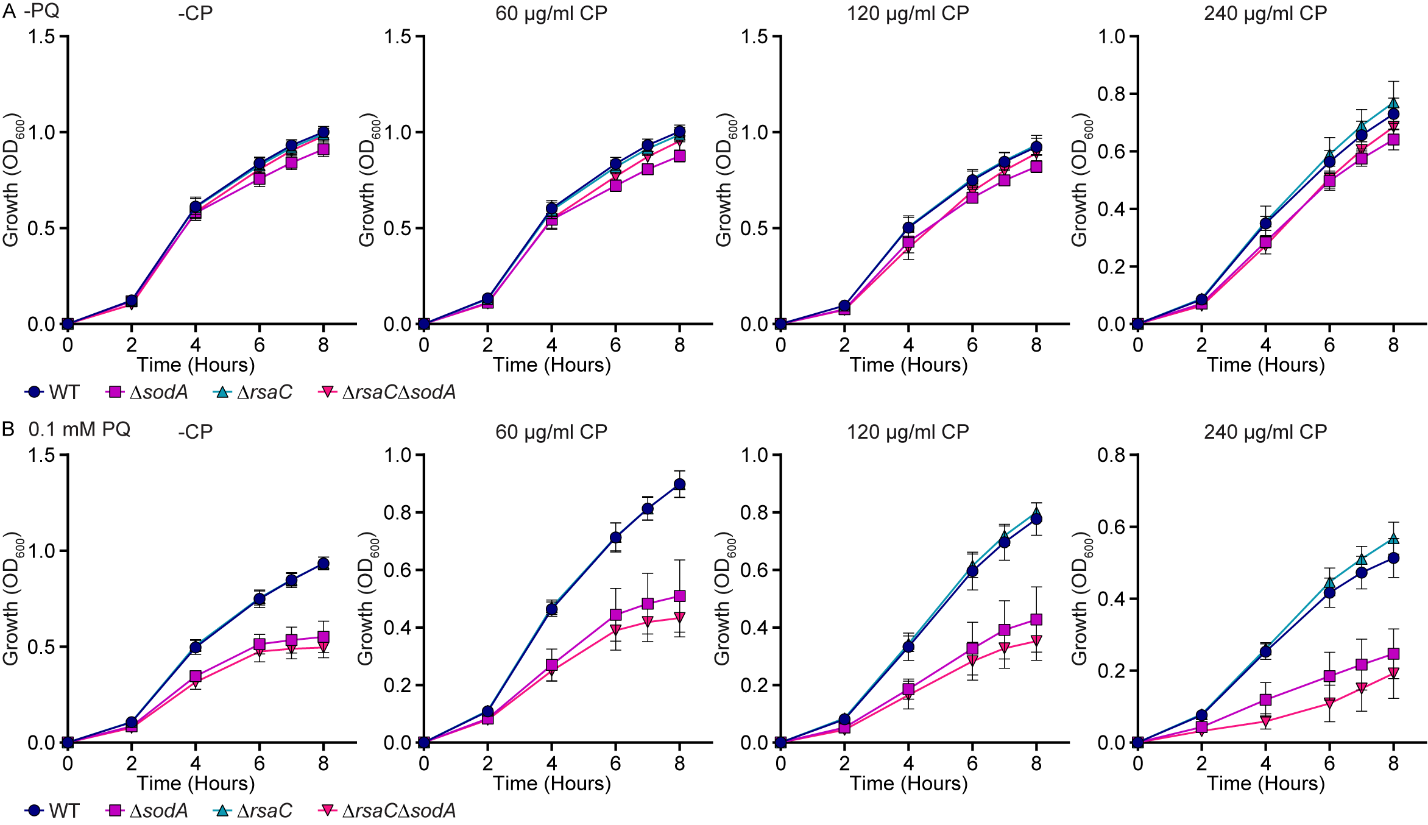


**Supplemental Figure 8.** **Growth curves in TSB with and without PQ across various CP concentrations.**

The indicated strains of *S. aureus* were incubated in TSB with CP in the (A) absence or (B) presence of 0.1 mM PQ and growth was assessed by measuring optical density over 8 h. n = 4. Error bars = SD.


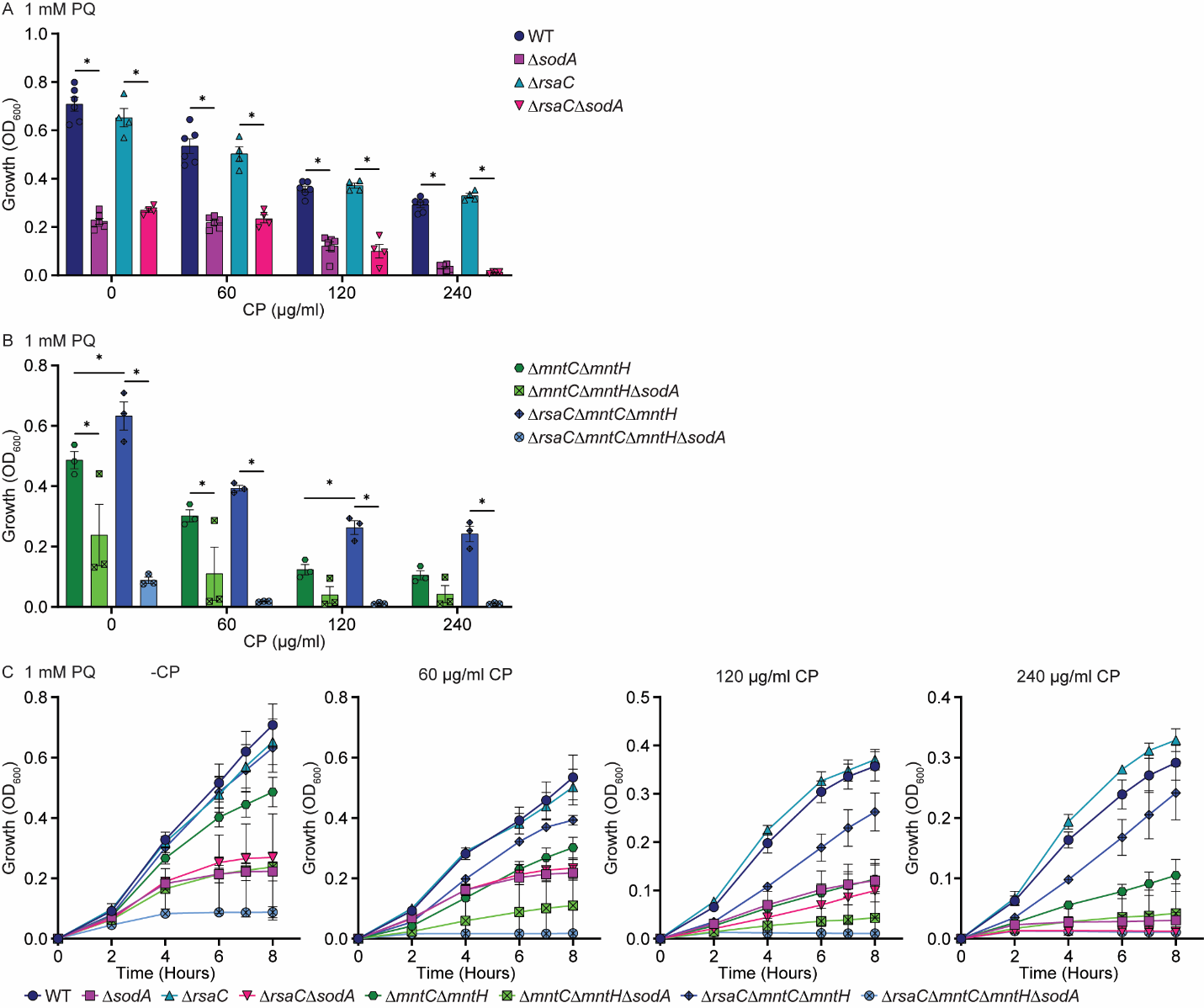


**Supplemental Figure 9.** **The impact RsaC’s suppression of SodA on oxidative stress sensitivity remains true at a higher PQ concentration and in the Mn transporter mutants.**

(A-C) The indicated strains of *S. aureus* were incubated in TSB in the presence of 1 mM PQ and CP and growth was assessed by measuring optical density over 8 h. (A, B) * = p $\leq$ 0.05 of the indicated comparison via two-way ANOVA with Dunnett’s multiple comparisons test. (A-C) n $\geq$ 3. (A-B) Error bars = SEM. (C) Error bars = SD.


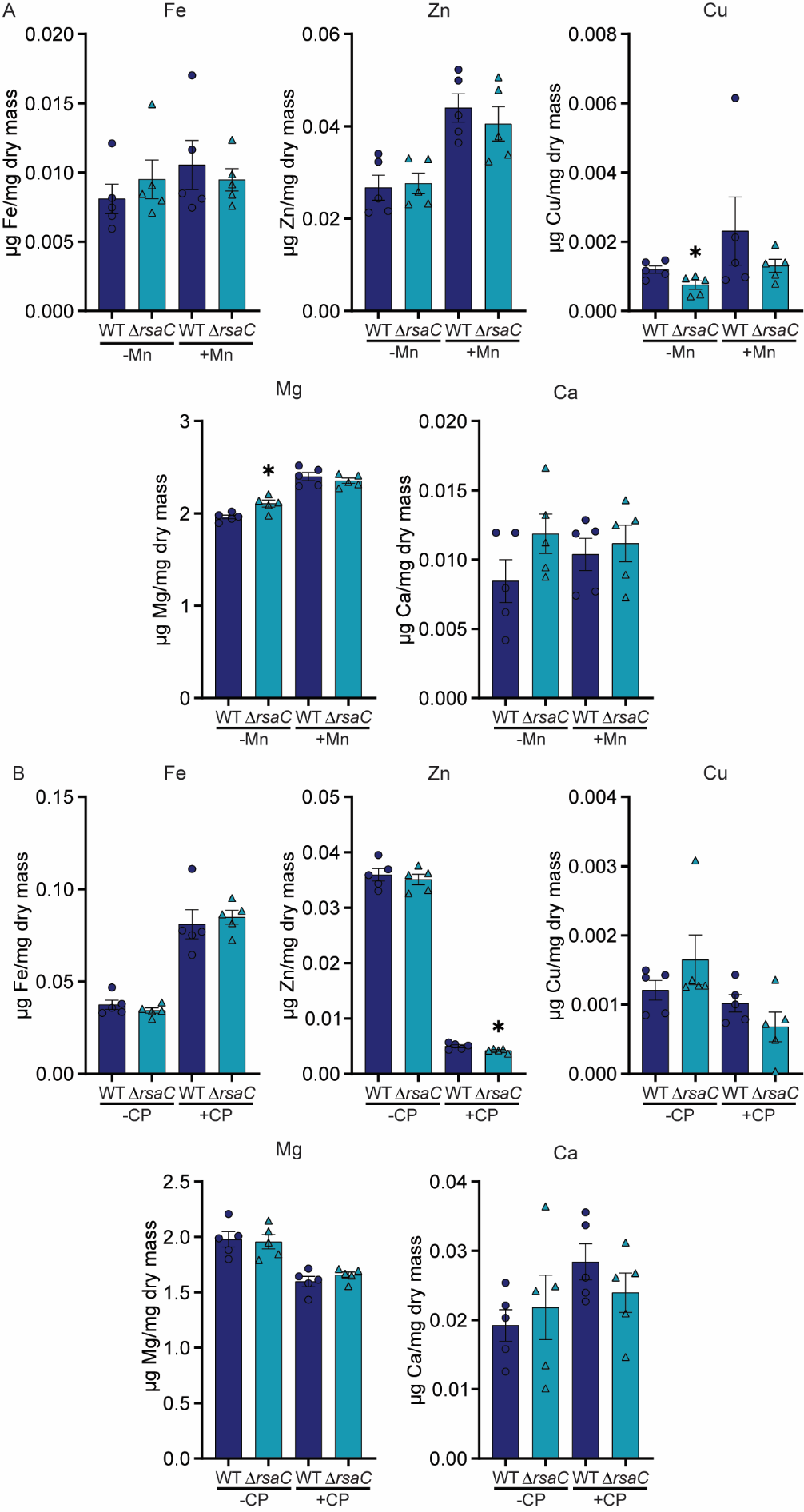


**Supplemental Figure 10. Loss of RsaC does not impact the intracellular concentration of other metals.**

Intracellular concentration of the indicated metals was measured in *S. aureus* wild type and Δ*rsaC* cells using ICP-OES following growth to an OD_600_ of ∼0.25 in either (A) NRPMI with and without Mn supplementation or (B) TSB in the presence and absence of 240 µg/mL CP. (A-B) * = p $\leq$ 0.05 of the indicated comparison via unpaired t test. Error bars = SEM.

**Supplemental Tables**

**Supplemental Table 1. *Staphylococcus aureus strains* used in this study.**

| **Bacterial strain** | **Genotype** | **Source** |
| --- | --- | --- |
| *Staphylococcus aureus* Newman | Wild type |  |
| Δ*rsaC* | *S. aureus* Newman Δ*rsaC* | This study |
| Δ*mntC*Δ*mntH* | Newman Δ*mntC*Δ*mntH* | (25) |
| Δ*rsaC*Δ*mntC*Δ*mntH* | Newman Δ*rsaC*Δ*mntC*Δ*mntH*:: *erm* | This study |
| Δ*mntC*Δ*mntH*Δ*sodA* | Newman Δ*mntC*Δ*mntH*Δ*sodA*:: *tet* | This study |
| Δ*rsaC*Δ*mntC*Δ*mntH*Δ*sodA* | Newman Δ*rsaC*Δ*mntC*Δ*mntH*::*erm*Δ*sodA*:: *tet* | This study |

**Supplemental Table 2. Primers used in this study.**

| **Primer** | **Sequence (5’3’)** |
| --- | --- |
| 5’ rsaC_F | gctatgaccatgtaatacgactcactataggggatgtagttgcacaagtattattgc |
| 5’ rsaC_R | aatagtcccatatcgtgggctattttttgtgaatacacttaac |
| 3’ rsaC_F | tattcacaaaaaatagcccacgatatgggactattagcc |
| 3’ rsaC_R | gaacgaccgagcgcagcgagtcagtgagcgaggagaggaagtttaacatgaaaaaattag |
| 5’ rsaC/mntC_F | gaccatgtaatacgactcactataggggataaaagcaaagcaatactttgatc |
| 5’ rsaC/mntC_R | caaaaaatagccacaactaattttttcatgttaaacttcctc |
| 3’ rsaC/mntC_F | catgaaaaaattagttgtggctattttttgtgaatacac |
| 3’ rsaC/mntC_R | gaccgagcgcagcgagtcagtgagcgaggagttgcacaagtattattgccattattaac |
| rsaC pOS1 p_lgt__F | gagtagggataaatacaattgaggtgaacacatatcgtgcgttaaatatatattacc |
| rsaC pOS1 p_lgt__R | aaaaagggggaaacactacccccttgtttgaaaatagccacactcatatgac |
| pCN52_F | atgagtaaaggagaagaacttttc |
| pCN52_R | atgtatatctccttcttaaattaattagttaac |
| pHELP_F | aactaattaatttaagaaggagatatacatatcccattatgctttggcag |
| pHELP_R | attattttctctactgggtttcactctccttctac |
| SodA TL (long)_F | aggagagtgaaacccagtagagaaaataataagtagtagaatttaag |
| SodA TL (long)_R | tccagtgaaaagttcttctcctttactcattgcgtatggtaattttggtaattc |

**Supplemental Table 3. Plasmids used in this study.**

| **Plasmid** | **Description** | **Source** |
| --- | --- | --- |
| pAH5: empty | pAH5 lacking a promoter for YFP expression | (33) |
| pAH5: *mntABC* | Plasmid for *mntABC* promoter-dependent YFP expression | (70) |
| pAH5: *sodA* | Plasmid for *sodA* promoter-dependent YFP expression | (33) |
| pCN52: empty | pCN52 lacking a promoter for GFP expression | (71) |
| pCN52: *sodA* | Plasmid for pHELP-driven *sodA* translation (including the first 11 codons of the gene) | This study |
| pKK30: kan | Stable plasmid with kanamycin cassette | (72) |
| pKK30: tet | Stable plasmid with tetracycline cassette | (72) |
| pOS1 P*_lgt_* : empty | Complementation plasmid containing the *lgt* promoter | (73) |
| pOS1 P*_lgt_* : *rsaC* | Complementation plasmid expressing RsaC under the control of *lgt* promoter | This study |

**REFERENCES**

70. Solórzano PKP, Yao J, Rock CO, Kehl-Fie TE. 2019. Disruption of glycolysis by nutritional immunity activates a two-component system that coordinates a metabolic and antihost response bysruption of Glycolysis by Nutritional Immunity Activates a Two-Component System That Coordinates a Metabolic and Antihost Response by Staphylococcus aureus. MBio 10:01321–19. https://doi.org/10:10.1128/mbio.01321-19

71. Charpentier E, Anton AI, Barry P, Alfonso B, Fang Y, Novick RP. 2004. Novel cassette-based shuttle vector system for gram-positive bacteria. Appl Environ Microbiol 70:6076–6085. <https://doi.org/10.1128/AEM.70.10.6076-6085.2004>

72.  Hossain S, Morey JR, Neville SL, Ganio K, Radin JN, Norambuena J, Boyd JM, McDevitt CA, Kehl-Fie TE. 2023. Host subversion of bacterial metallophore usage drives copper intoxication. MBio 14:e01350–23. https://doi.org/10.1128/mbio.01350-23

73. Schneewind O, Model P, Fischetti VA. 1992. Sorting of protein A to the staphylococcal cell wall. Cell 70:267–281. https://doi.org/10.1016/0092-8674(92)90101-h

**Supplemental Table 4. Recipe for carbon source defined media.**

| **Reagent** | **Concentrated Media** | **1 X Media** |
| --- | --- | --- |
| NaCl | 1.3 g/liter | 0.494 g/liter |
| NH_4_Cl | 2.6 g/liter | 0.988 g/liter |
| KH_2_PO4 | 5.2 g/liter | 1.976 g/liter |
| Na_2_HPO4 | 18.2 g/liter | 6.916 g/liter |
| biotin | 0.593 µg/liter | 0.22534 µg/liter |
| nicotinic acid | 0.593 mg/liter | 0.22534 mg/liter |
| pyridoxine-HCl | 0.593 mg/liter | 0.22534 mg/liter |
| thiamine-HCl | 0.593 mg/liter | 0.22534 mg/liter |
| riboflavin | 0.296 mg/liter | 0.11248 mg/liter |
| calcium pantothenate | 1.778 g/liter | 0.67564 g/liter |
| phenylalanine | 0.104 g/liter | 0.03952 g/liter |
| isoleucine | 0.078 g/liter | 0.02964 g/liter |
| tyrosine | 0.13 g/liter | 0.0494 g/liter |
| cysteine | 0.053 g/liter | 0.02014 g/liter |
| glutamic acid | 0.26 g/liter | 0.0988 g/liter |
| lysine | 0.026 g/liter | 0.00988 g/liter |
| methionine | 0.182 g/liter | 0.06916 g/liter |
| histidine | 0.078 g/liter | 0.02964 g/liter |
| tryptophan | 0.026 g/liter | 0.00988 g/liter |
| leucine | 0.234 g/liter | 0.08892 g/liter |
| aspartic acid | 0.234 g/liter | 0.08892 g/liter |
| arginine | 0.182 g/liter | 0.06916 g/liter |
| serine | 0.078 g/liter | 0.02964 g/liter |
| alanine | 0.15 g/liter | 0.057 g/liter |
| threonine | 0.078 g/liter | 0.02964 g/liter |
| glycine | 0.130 g/liter | 0.0494 g/liter |
| valine | 0.208 g/liter | 0.07904 g/liter |
| proline | 0.026 g/liter | 0.00988 g/liter |
| Casamino Acids (as indicated) | 6.5% | 2.47% |
| Glucose (as indicated) | 1.3% | 0.494% |
